# Novel metabolic features in *Acinetobacter baylyi* ADP1 revealed by a multiomics approach

**DOI:** 10.1007/s11306-014-0662-x

**Published:** 2014-04-29

**Authors:** Lucille Stuani, Christophe Lechaplais, Aaro V. Salminen, Béatrice Ségurens, Maxime Durot, Vanina Castelli, Agnès Pinet, Karine Labadie, Stéphane Cruveiller, Jean Weissenbach, Véronique de Berardinis, Marcel Salanoubat, Alain Perret

**Affiliations:** 1 Direction des Sciences du Vivant, Commissariat à l’Energie Atomique et aux Energies Alternatives (CEA), Institut de Génomique, Evry, France; 2 CNRS-UMR8030, Evry, France; 3 Université d’Evry Val d’Essonne, Evry, France; 4 Department of Chemistry and Bioengineering, Tampere University of Technology, Korkeakoulunkatu 10, 33720 Tampere, Finland

**Keywords:** LC/MS-LTQ-Orbitrap, Metabolomics, Transcriptomics, Functional genomics, Bacterial metabolism

## Abstract

Expansive knowledge of bacterial metabolism has been gained from genome sequencing output, but the high proportion of genes lacking a proper functional annotation in a given genome still impedes the accurate prediction of the metabolism of a cell. To access to a more global view of the functioning of the soil bacterium *Acinetobacter baylyi* ADP1, we adopted a multi ‘omics’ approach. Application of RNA-seq transcriptomics and LC/MS-based metabolomics, along with the systematic phenotyping of the complete collection of single-gene deletion mutants of *A. baylyi* ADP1 made possible to interrogate on the metabolic perturbations encountered by the bacterium upon a biotic change. Shifting the sole carbon source from succinate to quinate elicited in the cell not only a specific transcriptional response, necessary to catabolize the new carbon source, but also a major reorganization of the transcription pattern. Here, the expression of more than 12 % of the total number of genes was affected, most of them being of unknown function. These perturbations were ultimately reflected in the metabolome, in which the concentration of about 50 % of the LC/MS-detected metabolites was impacted. And the differential regulation of many genes of unknown function is probably related to the synthesis of the numerous unidentified compounds that were present exclusively in quinate-grown cells. Together, these data suggest that *A. baylyi* ADP1 metabolism involves unsuspected enzymatic reactions that await discovery.

**Electronic supplementary material:**

The online version of this article (doi:10.1007/s11306-014-0662-x) contains supplementary material, which is available to authorized users.

## Introduction

Extensive insights into the metabolism of microbial organisms have been gained from whole genome sequencing and annotation projects. Nevertheless, with approximately 30 % of genes of a typical genome with no assigned function, the genome mining alone cannot report for the vast diversity in the lifestyle and metabolic functions observed in the bacterial world. To this end, functional genomics, which aims at the elucidation of the molecular basis of biological functions, requires analyses that go far beyond the primary analysis of the genome sequence. Technologies such as phenomics, transcriptomics, and metabolomics are complementary tools for exploring the metabolic state of microorganisms, allowing a more global view of the functioning of a cell. *Acinetobacter* spp. are gram-negative bacteria that are ubiquitously distributed in nature. Unique among this highly heterogeneous bacterial genus (Ibrahim et al. 1997) is strain ADP1, a soil bacterium characterized by a small genome (3.6 Mb) in which genes encoding most catabolic functions are clustered in several genetic islands (Barbe et al. 2004; Young et al. 2005). Its extraordinary competence for natural transformation and the ease with which it can be genetically engineered (de Berardinis et al. 2009; Metzgar et al. 2004) make ADP1 a key organism for the study of metabolism.

**Fig. 1:**
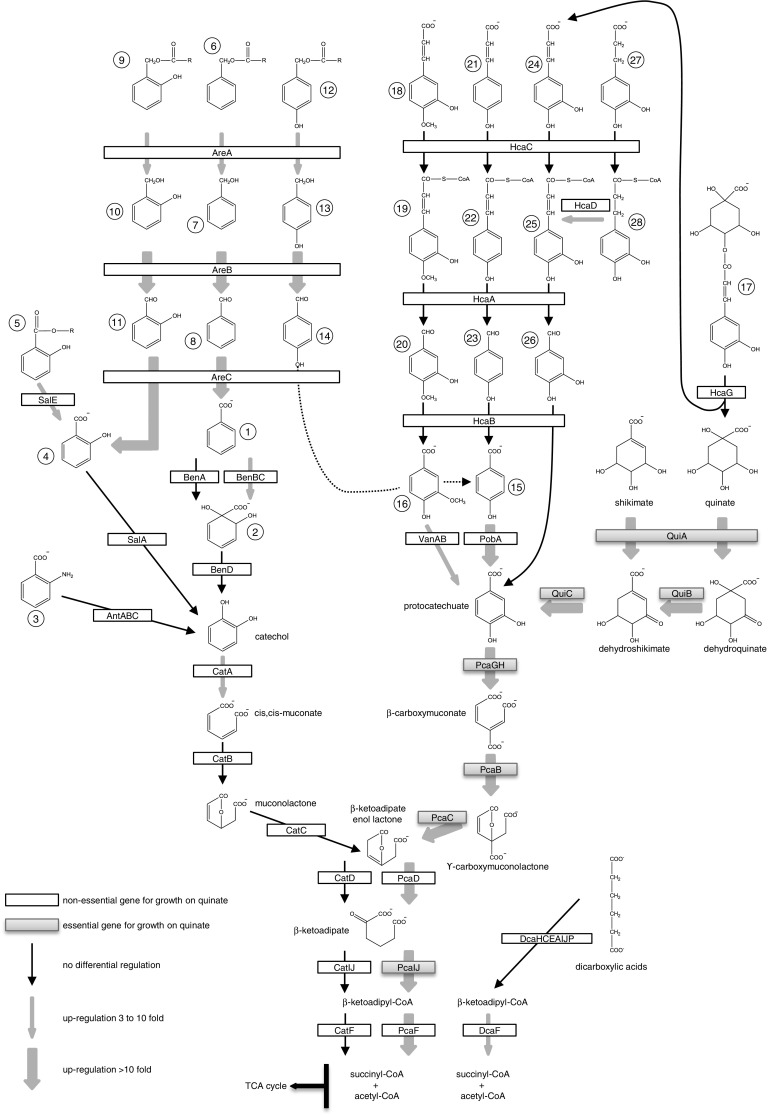
The complete pathway for aromatic catabolism in *A. baylyi* ADP1. Enzymes are labeled within boxes by their genetic notation. Unnamed metabolites are labeled with circled numbers: *1* benzoate, *2* 1,2-dihydro-1,2-dihydroxybenzoate (benzoate *cis*-glycol), *3* anthranilate, *4* salicylate, *5* alkyl salicylates, *6* benzylalkanoates, *7* benzyl alcohol, *8* benzaldehyde, *9* 2-hydroxybenzylalkanoates (salicylalkanoates), *10* 2-hydroxybenzyl alcohol, *11* 2-hydroxybenzaldehyde, *12* 4-hydroxybenzylalkanoates, *13* 4-hydroxybenzyl alcohol, *14* 4-hydroxybenzaldehyde, *15* 4-hydroxybenzoate (*p*-hydroxybenzoate), *16* vanillate, *17* chlorogenate, *18* ferulate, *19* ferulyl-CoA, *20* vanillaldehyde, *21* *p*-coumarate, *22 p*-coumaryl-CoA, *23* 4-hydroxybenzaldehyde, *24* caffeate, *25* caffeyl-CoA, *26* protocatechualdehyde, *27* 4-hydroxyphenylpropionate, *28* 3,4-dehydroxyphenylpropionyl-CoA. Adapted from (Williams and Kay 2008)

ADP1 is a nutritionally versatile bacterium capable of metabolizing a wide range of aromatic compounds as the sole source of carbon and energy (Barbe et al. 2004). The main route for the degradation of aromatic molecules to the point where they can enter central metabolism is the β-ketoadipate pathway through catechol and protocatechuate (Young et al. 2005). The main features of the β-ketoadipate pathway have been described in detail (Harwood and Parales 1996). From catechol and protocatechuate, a parallel but separate branch converts them into succinyl-CoA and acetyl-CoA which can enter central metabolism through the TCA cycle (Fig. 1). Most of the genes involved in the degradation of aromatic compounds that feed into the catechol branch are colocalized with those for catechol degradation, forming one island of catabolic genes. This cluster contains the sal-are-ben-cat genes (ACIAD1424 to ACIAD1451). On the other hand, most of the genes that metabolize aromatic substrates feeding into the protocatechuate branch are colocalized with those for protocatechuate degradation and form another island of catabolic genes, which contains the pca-qui-pob-hca genes (ACIAD1702 to ACIAD1728).

Quinate is an alicyclic compound that feeds into the protocatechuate branch. Its conversion to protocatechuate takes place in the periplasm, where quinate dehydrogenase (QuiA) oxydates quinate to dehydroquinate, which is then dehydrated by dehydroquinate dehydratase (QuiB) to produce dehydroshikimate. This latter is further dehydrated by dehydroshikimate dehydratase (QuiC) to produce protocatechuate which is then translocated in the cytoplasm for further degradation (Young et al. 2005). Quinate dissimilation is performed by 14 genes, 3 code for 2 transporters and 1 regulator, the remaining being involved in enzymatic activities. In sum, the biochemical and genetic features of quinate degradation in ADP1 have been thoroughly investigated for many years (for review, see Young et al. 2005). In contrast, the question on the genes and enzymes activated by quinate and how they are differentially regulated, at the genome scale, remains largely unanswered.

In this work, we have adopted a multi ‘omics’ approach for interrogating on the metabolic perturbations encountered by ADP1 when the sole source of carbon and energy shifted from succinate to quinate. To this end, we reexamined the systematic phenotyping of our collection of approximately 2,400 deletion mutants (de Berardinis et al. 2008) on liquid medium containing quinate as the sole carbon source, for detecting the genes involved in its catabolism. And for the first time, we report the use of RNA-seq transcriptomics and LC/MS-based metabolomics for exploring the metabolic response of ADP1 to face this environmental change. The comparison of expression profiles in quinate versus those in succinate confirmed the participation of the genes known to dissimilate quinate, but especially revealed a major reorganization of the transcription pattern of ADP1. This led in turn in a dramatic change in the metabolome, in which about 50 % of intracellular LC/MS-detected metabolites had their concentration changed. Together, these results indicated that the carbon source shift did not only induce the specific transcriptional response necessary to face the environmental change (i.e. the catabolism of the new carbon source) but also suggested a more global metabolic reprogramming. Finally, the differential regulation of many genes of unknown function along with the accumulation of metabolites of unknown identity suggest that unsuspected metabolic pathways take place during growth on quinate.

## Materials and methods

### Chemicals and reagents

Agarose (Seakem^®^ GTG™) was purchased from Lonza. PTFE membrane filters (JH Omnipore 0.45 µm) were from Millipore. Cryogenic vials (T3082A) were from Simport. HPLC-grade solvents, ammonium acetate, ammonium carbonate, ammonium hydroxide, formic acid, and metabolite standards were from Sigma-Aldrich. 3-dehydroquinate was prepared from quinate and cell crude lysate containing quinate dehydrogenase (see Online Resource 1, Protocol S-1).

### Strain and media

The *A. baylyi ADP1* strain (DSM 24193) was provided by Dr. Nicholas Ornston (Yale University). Cells were routinely grown on MA (Medium for *Acinetobacter*) minimal medium [31 mM Na_2_HPO_4_, 25 mM KH_2_PO_4_, 18 mM NH_4_Cl, 41 mM nitrilotriacetic acid, 2 mM MgSO_4_, 0.45 mM CaCl_2_, 3 mM FeCl_3_, 1 mM MnCl_2_, 1 mM ZnCl_2_, 0.3 mM (CrCl_3_, H_3_BO_3_, CoCl_2_, CuCl_2_, NiCl_2_, Na_2_MoO_2_, Na_2_SeO_3_)] supplemented with 25 mM of the desired carbon source.

### Construction of the deletion mutants

PCR primers are listed in Online Resource 1, Table S-1. Generation of the integration cassette, transformation and mutant selection were conducted as previously reported (de Berardinis et al. 2008). ΔACIAD3353 and ΔACIAD1738 were first selected on MA supplemented with 25 mM succinate and 30 µg/ml kanamycine, and containing 25 mM shikimate to allow aromatic amino acid biosynthesis. The clones were then replicated on MA supplemented with succinate and on MA supplemented with quinate.

### RNA-seq transcriptomics

#### Culture conditions for RNA preparation

Duplicate liquid cultures of ADP1 were grown at 30 °C in MA minimal medium supplemented with 25 mM of each of the desired carbon source and shaken at 150 rpm. Overnight cultures were used to inoculate (1:100 dilution) 100 ml of fresh medium. Cultures were grown at 30 °C and 150 rpm to a final OD of 0.6–0.7 at 600 nm (mid-log phase), and stopped by adding 12.5 ml of RNA stabilization buffer (2.5 % phenol-acetate 25 mM pH 5.5 + 95 % ethanol). Bacteria were collected by centrifugation (4 °C for 3 min at 6,000 g) and suspended in 20 ml of Tri Reagent^®^ (Sigma-Aldrich) for RNA extraction and stored at −20 °C. Total RNA was isolated according to the Tri Reagent^®^ supplier protocol. Residual genomic DNA was removed using Ambion DNase Turbo^®^ (Life Technologies) and DNA degradation was checked by PCR with primers designed for selected ORFs to measure the contamination of residual genomic DNA. RNA integrity was analyzed using an Agilent 2100 bioanalyzer (Agilent technologies, Santa Clara, CA, USA). Ribosomal RNA was removed by Ambion MICROBExpress^®^ and MEGAclear^®^ treatments. To evaluate the degree of rRNA depletion, samples were analyzed on an Agilent 2100 bioanalyzer. The RNA Integrity Number (RIN) of total RNA was >7.

#### Fragmentation of RNA

0.5–1 µg RNA was fragmented to size between 100 and 200 nt using RNA Fragmentation Buffer (5×: 200 mM Tris/acetate, pH 8.1, 500 mM KOAc, 150 mM MgOAc), based on metal-catalyzed heat fragmentation. 4 µl of 5× RNA Fragmentation Buffer were added to 16 µl of RNA and incubated for 3 min at 94 °C. The reaction was terminated by adding 2 µl of Stop Solution (containing a metal chelating agent) and chilled on ice. RNA was then purified on a RiboMinus™ Concentration Module (Life Technologies). The fragmented RNA was dephosphorylated using 2 U Antarctic phosphatase (New England Biolabs) at 37 °C for 30 min, and 5′phosphorylated using 20 U T4 polynucleotide Kinase (New England Biolabs) at 37 °C for 60 min. The reaction product was purified with the RiboMinus™ Concentration Module.

#### 3′ adapter ligation

The 3′ adapter was ligated to the RNA fragments as follows: 5 µl mRNA (0.5–1 µg) and 1 µl 3′ adapter (100 µM) were pre-heated at 70 °C for 2 min and immediately placed on ice, to minimize secondary structure formation. 2 µl 5× HM Ligation Buffer (supplied with Illumina TruSeq Small RNA Sample Prep Kit) were added with 1 µl RNasin^®^ RNase inhibitor (Promega) and 200 U T4 RNA Ligase 2, truncated (New England Biolabs). The reaction was performed for 1 h at 28 °C followed by 15 min at 28 °C in the presence of 1 µl of stop solution (STP) and placed on ice to inactivate the enzyme.

#### 5′ adapter ligation

The 5′ adapter was ligated as follows: 1 µl of 5′ adapter (100 µM) was pre-heated at 70 °C for 2 min and chilled on ice. The pre-heated 5′ adapter was then added to the 3′ adapter ligation reaction product in the presence of 1 µl ATP (10 mM) and 20 U of T4 RNA Ligase 1 (New England Biolabs). The ligation reaction was performed for 1 h at 28 °C and chilled on ice.

#### First strand reverse transcription and PCR amplification

Six microlitre of the 5′ and 3′ adapter-ligated RNA and 1 µl of the Illumina RT primer (100 µM) were pre-heated at 70 °C for 2 min and placed on ice. Next, 2 µl 5× First-Strand Buffer (250 mM Tris/HCl pH 8.3, 375 mM KCl, 15 mM MgCl_2_; Invitrogen), 0.5 µl 12.5 mM dNTP, 1 µl 100 mM DTT, 1 µl RNasin^®^ RNase inhibitor, and 200 U of SuperScript™ II reverse transcriptase (Invitrogen) were added and allowed the first strand synthesis to proceed for 1 h at 50 °C and was then chilled on ice. The first strand was amplified in a PCR reaction containing 12.5 µl of ssDNA, 10 µl 5× Phusion^®^ HF Buffer (New England Biolabs), 2 µl RNA PCR Primer (RP1 supplied with Illumina TruSeq Small RNA Sample Prep Kit), 2 µl RNA PCR Primer Index (RPlX containing a unique 6-bases index sequence), 0.5 µl 25 mM dNTP and 0.5 µl of Phusion^®^ High-Fidelity DNA polymerase. The PCR protocol used for the libraries was as follows: 30 s, 98 °C/12× (10 s, 98 °C/30 s, 60 °C/15 s, 72 °C)/10 min, 72 °C/hold at 4 °C. Libraries were purified using AMPure beads (Agencourt Bioscience) according to the Directional mRNA-seq library preparation v1.0.

#### DNA sequencing

Libraries were purified and then quantified using a Qubit Fluorometer (Life technologies) and libraries profiles were evaluated using an Agilent 2100 bioanalyzer (Agilent Technologies, USA). Libraries were loaded at a concentration of 12 pM per flow cell lane and sequenced on the Illumina GAIIx instrument using 76 base-length read chemistry in a single flow cell, following manufacturer instructions. Image analysis and base calling were performed using Illumina pipeline v 1.6.

#### RNA-Seq analysis

Transcriptomic high throughput sequencing data were analyzed using a bioinformatic pipeline implemented in the Microscope platform (Vallenet et al. 2013). The current pipeline was a “Master” shell script that launches the various parts of the analysis (i.e. a collection of Shell/Perl/R scripts) and controls for all tasks having been completed without errors. In a first step, the RNA-seq data quality was assessed by including option like reads trimming or merging/split paired-end reads. In a second step, reads were mapped onto ADP1 genome sequence (GenBank accession no CR543861) using the SSAHA2 package (Ning et al. 2001) that combined the SSAHA searching algorithm (sequence information is encoded in a perfect hash function) aiming at identifying regions of high similarity, and the cross-match sequence alignment program (Ewing et al. 1998), which aligned these regions using a banded Smith–Waterman–Gotoh algorithm (Smith and Waterman 1981). An alignment score equal to at least half of the read was required for a hit to be retained. To lower false positives discovery rate, the SAMtools (v.0.1.8) (Li et al. 2009) were then used to extract reliable alignments from SAM formatted files. The number of reads matching each genomic object harbored by the reference genome was subsequently computed with the Bioconductor-GenomicFeatures package (Carlson 2011). If reads matching several genomic objects, the count number was weighted in order to keep the same total number of reads. Finally, the Bioconductor-DESeq package (Anders and Huber 2010) with default parameters was used to analyze raw counts data and test for differential expression between conditions.

Genes were considered up-regulated relative to succinate if the number of corresponding reads increased at least threefold (padj < 0.05). Conversely, genes were considered down-regulated if the number of reads decreased at least threefold (padj < 0.05).

### Metabolomics

#### Metabolome preparation

Six independent cultures were grown on each carbon source (the experimental design is described in Online Resource 1, Figure S-1). Metabolite extraction was adapted from (Brauer et al. 2006). A saturated overnight minimal media liquid culture was diluted in a fresh liquid media at an OD_600_ = 0.05, and further grown to an OD_600_ = 0.2. 5 ml of this culture were filtered onto a 47 mm PTFE filter (0.45 µm). The filter was then positioned with cells on top on an agarose plate containing the same minimal media. Cells were grown to log phase (OD_600_ ~ 0.8). Metabolism was quenched by placing the filter containing the cells face-down in a glass dish containing 5 ml of a cold (−30 °C) mixture of 80 % acetonitrile and 20 % methanol. After 15 min at −30 °C, the filter coated with cells was submitted to a mild sonication in an ultrasonic bath (Bronson 2510 Ultrasonic Cleaner) for 5 min at 4 °C to remove the cells from the filter. The quenching liquid containing the cells was transferred into a cryogenic vial and underwent 6 freeze/thaw cycles in liquid nitrogen/65 °C water to fully break the cells and extract the metabolites. The samples were lyophilized and first dissolved in 300 µl water. The debris were removed by centrifugation (2,000 g, 4 °C, 10 min) and the supernatant was filtered on 0.22 µm (Millipore Millex-GV 13 mm). The filter was further washed with 700 µl 80/20 acetonitrile containing 10 mM ammonium carbonate (pH adjusted to 9.9 with NH_4_OH). The resulting 1 ml samples were stored at 4 °C and analyzed within 24 h.

#### Chromatographic conditions

Analyses were conducted using an Accela LC system (Thermo Fisher Scientific, Courtaboeuf, France) with two different chromatographic columns. Chromatographic separation using an Acquity^®^ C18 column (150 × 2.1 mm^2^; 1.7 µm; Waters) was carried out at 50 °C as follow: a mobile phase gradient was used with a flow rate of 0.4 ml/min in which mobile phase A consisted of 10 mM ammonium acetate with pH adjusted to 3.5 with 0.1 % (vol/vol) formic acid and mobile phase B consisted of methanol. The gradient started at 100 % A for 1 min, followed by a linear gradient at 100 % B for 9 min, and remained 8 min at 100 % B. The system returned to the initial solvent composition in 2 min and re-equilibrated under these conditions for 5 min. Elution from a ZIC^®^-*p*HILIC column (150 × 4.6 mm^2^; 5 µm; Merck Sequant) was conducted at 40 °C using a mobile phase gradient with a flow rate of 0.5 ml/min. A consisted of 10 mM ammonium carbonate with pH adjusted to 9.9 with NH_4_OH and B of acetonitrile. The gradient started at 80 % B for 2 min, followed by a linear gradient to 40 % B at 22 min and remained 8 min at 40 % B. The system returned to the initial solvent composition in 5 min and re-equilibrated under these conditions for 15 min. For each chromatographic method, the autosampler was kept at 4 °C and 10 µl were injected.

#### LTQ-Orbitrap analysis

High-resolution measurements were obtained with a LTQ-Orbitrap mass spectrometer (Thermo Fisher Scientific) fitted with a heated electrospray ionisation source (HESI) operating in positive and negative ionization modes. In the positive ion mode, the electrospray voltage was set to 4.5 kV, the capillary voltage to 48 V, and the tube lens offset to 80 V. The sheath and auxiliary gas flows (both nitrogen) were respectively optimized at 60 and 50 (arbitrary units), and the drying gas temperature was set to 275 °C. The same settings were used for the negative ion mode, except for the electrospray voltage, which was fixed at 4 kV, and the capillary voltage and tube lens offset, which were set to −30 and −90 V, respectively. Mass spectra were acquired over an *m*/*z*-range from *m*/*z* 50 up to *m*/*z* 1000 with the mass resolution set to 30,000 FWHM at *m*/*z* 400 in the Orbitrap analyzer. Collision-induced dissociation spectra (CID) in resonant excitation conditions were acquired using data-dependent scanning function for identification purpose. Target gas for CID was helium. The scan event cycle comprised a mass spectrum at a resolution power of 30,000 and data-dependent (MS^2^) events acquired with the ion trap. Microscan count was set to unity and a repeat count for dynamic exclusion was set to 3. MS^n^ acquisition parameters were an isolation width of *m*/*z* 1, normalized collision energy at 35 %, and an activation time of 30 ms.

#### Preparation of standards and calibration solutions

Commercial metabolite standards were prepared for each analytical run from a 0.1 M solution in water stored at −20 °C and diluted to 50 µM in 80/20 acetonitrile/ammonium carbonate pH 9.9 for ZIC^®^-*p*HILIC analysis and in 80/20 ammonium acetate pH 3.5/methanol for C18 analysis. For ZIC^®^-*p*HILIC analysis, calibration curves of AMP, ADP and ATP were required to calculate the Adenylate Energy Charge (see below); we prepared calibration solutions with concentrations of 0.1, 0.5, 1, 2.5, 5, 7.5, 10 µM for AMP and ADP and with concentrations of 2, 5, 10, 15, 20 µM for ATP in 30 % water and 70 % mobile phase containing 80/20 acetonitrile/ammonium carbonate pH 9.9. Calibration curves were done in positive ionization mode (Online Resource 2, Table S-2). The matrix effect on the AEC calculation in ADP1 metabolomic samples was investigated. The use of ^15^N labelled nucleotides revealed only a slight underestimation of the AEC (data not shown).

#### Metabolomic data processing

LC/MS data were acquired in raw files and processed with the Qualbrowser module of Xcalibur 2.2 (Thermo Fisher Scientific) to access to elemental compositions. 2 biological replicates originating from the same quinate cultures day (Online Resource 1, Figure S-1) were removed from the data set due to technical problems. This led to a reduced data set consisting of 6 and 4 metabolomes originating from succinate- and quinate-grown cells, respectively. Each metabolome was injected twice and considered as analytical replicates. This data set was thus used as the raw data for data pretreatment. For global analysis, raw data files were converted into the mzXML format using the MassMatrix File Conversion Tools (http://www.massmatrix.net). Data were analyzed by the web-based platform XCMS Online (Tautenhahn et al. 2012) version 1.22.01. The centWave algorithm (Tautenhahn et al. 2008) was used. The parametring of XCMS was conducted in both ionization modes with a mixture of 126 commercial compounds representative of the metabolism, at a concentration of 50 µM each. Out of them, 119 were detected in the RAW data (not shown). We first used HPLC/Orbitrap II default settings and adjusted empirically the parameters to optimize the detection of the standards. The parameters that were tuned were *ppm*, *max peakwidth*, *prefilter intensity*, *noise filter*, and *minfrac*, which were respectively set at 3, 45, 100, 0, and 0.75. *S/N threshold* was set at 6 and 3 for the positive and negative ionization modes, respectively. Using this parametring, 117 out of the 119 standards detected in the RAW files could be identified by XCMS (although 7 were poorly detected). This refined parametring was used to process the ADP1 metabolomic data. Redundant ion signals (natural isotopes, adduct ions, dimer ions and fragment ions) were automatically annotated and combined in different feature groups of related peaks, each group corresponding to one potential metabolite. A final data set was defined after manual verification in the raw data of each peak detected by XCMS, in both ionization modes, to remove ions also present in the medium culture, misintegrated peaks, and all redundant information. The analytical reproducibility was determined by establishing the relative standard deviation (RSD) between the analytical replicates. The biological reproducibility, i.e. the variability of the concentration of a specific metabolite as determined in ADP1 cells grown under identical conditions in independent cultures, was also estimated by the RSD. When biological RSD calculations were ≥40 %, peaks area were manually verified and corrected, especially for peaks close to the detection limit. The selected mass chromatograms were putatively identified by matching the masses (mass accuracy <10 ppm) to those from public databases (KEGG and Metlin). Metabolite identification was aided by MS^n^ interpretation and retention time matching to commercial reference standards.

The concentration of a metabolite was considered as increased relative to succinate if it increased at least threefold in signal intensity (*P* < 0.05). Conversely, the concentration of a metabolite was considered as decreased for a reduction in signal intensity of at least threefold (*P* < 0.05). Using the R package Muma (Gaude et al. 2013), the final data set was first mean-centered, reduced and scaled to Pareto variance to perform principal component analysis (PCA).

#### Metabolite identification

Metabolites extracted from ADP1 were identified by comparison of the retention time, *m*/*z* ratio and MS^n^ spectra with those of authentic reference compounds, when available, from our in-house chemical library and from the one kindly provided by Dr. Christophe Junot.

## Results and discussion

As cell cultures for RNA-seq analysis were conducted in liquid medium, and those for metabolome extraction on solid medium (see Sects. 2, 2.5.1), growth kinetics in liquid medium and in filter cultures (with OD_600_ measurements done every 30 min) were conducted and compared. Results indicated that the growth behavior of ADP1 was similar in the two conditions (data not shown).

### RNA_seq analysis

#### Quinate triggered a large scale transcriptional reorganization

**Fig. 2:**
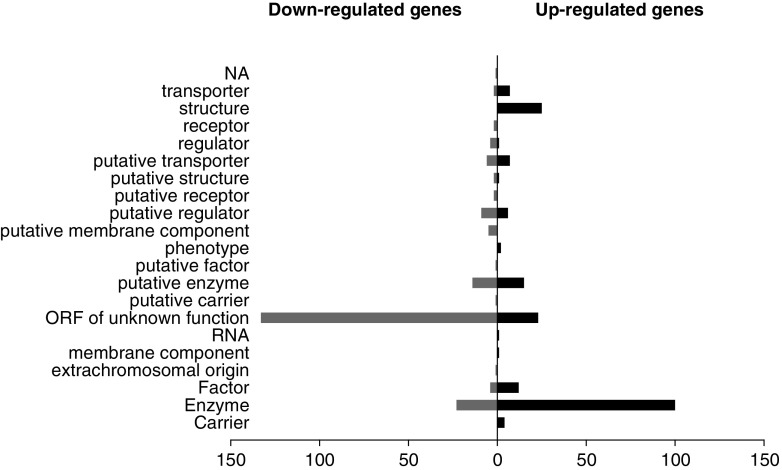
Number and functional classification of genes differentially expressed in *A. baylyi* ADP1 grown on quinate as compared with succinate. Each* plot* indicates the type of physiological role(s) and the total number of genes with increased or decreased expression within that category in cells grown on quinate (see also Online Resource 2, Table S-4)

High-throughput sequencing of cDNA prepared from RNA, a methodology known as RNA-seq, has become the method of choice for genome-wide transcriptome analyses, with unprecedented sensitivity and dynamic range (Wang et al. 2009; Wilhelm et al. 2010). Here, directional RNA sequencing libraries were prepared in duplicates from succinate and quinate-grown cells. A summary of the outcome of these experiments is presented in Online Resource 1, Table S-3. After mapping the reads on ADP1 genome sequence, data analysis yielded the relative expression level for 3,303 genes (Online Resource 2, Table S-4). The comparative analysis revealed that shifting the carbon source from succinate to quinate did not merely lead to the up-regulation of the genes implicated in the degradation of quinate, but altered the global transcription pattern of the cell (Fig. 2). More than 400 genes of different functional classes were affected, with an equivalent proportion of genes up- and down-regulated. Up-regulated genes mostly coded for enzymes (100), ribosomal proteins (25), proteins of unknown function (23) and proteins known to be involved in stress conditions (DnaK, DnaJ, GroES, GroEL, HslO, HtpG…). But the most unexpected observation was that the large majority of down-regulated genes were ORFs of unknown function (133). For most of them, their transcription status was not affected during heat shock at 42 °C (de Berardinis V., personal communication). It was reported that changing the nutritional regime of a bacterium could affect gene regulation (Klumpp et al. 2009). In particular, the number of genes differentially expressed increased as the quality of the carbon source declined (defined by the value of the growth rate). It can represent up to ~7 % (292 genes with a fold change ≥3) of the total number of genes in *E. coli* (Liu et al. 2005) and up to ~18 % (1,024 genes with a fold change ≥2) in *Pseudomonas putida* (Kim et al. 2013). For ADP1, both carbon sources used in this study were of similar quality, as succinate led to a growth rate of 0.84 ± 0.03 h^−1^ and quinate 0.79 ± 0.01 h^−1^ (values correspond to the average and standard deviation of three independent experiments). While in *P. putida* the use of carbon sources of similar quality affected ~6 % (325 genes with a fold change ≥2) of the genes (Kim et al. 2013), here, ~12 % (415 genes with a fold change ≥3) or ~30 % (1,086 genes with a fold change ≥2) of the genes were affected. In conclusion, our data indicate that quinate elicited not only a specific transcriptional response that allowed quinate dissimilation, but also a global transcriptional response that was independent of the growth rate of the cell.

#### Regulation of the protocatechuate branch of the β-ketoadipate pathway

As anticipated, the 14 genes known to participate in quinate degradation were among the most up-regulated (Fig. 1; Online Resource 2, Table S-4), further confirming that the protocatechuate branch of the β-ketoadipate pathway has to be induced for quinate dissimilation. The high expression level of the *pca* and *qui* genes observed here is consistent with an operonic organization of the *pcaI*-*quiA* region (Dal et al. 2005), and the carbon source-dependent regulation of *pcaU* expression observed here (13-fold) is also in agreement with previous results (Siehler et al. 2007). The protocatechuate branch of the β-ketoadipate pathway is under the transcriptional control of the activators-repressors PcaU and PobR. PcaU controls two promoters facing in opposite directions: the promoter for its own expression and the one for the expression of the first structural gene *pcaI* (Online Resource 1, Figure S-2A). PobR responds to *p*-hydroxybenzoate and activates the transcription of *pobA*, coding for the enzyme that converts *p*-hydroxybenzoate to protocatechuate. This latter triggers in turn the action of PcaU. Earlier studies indicated that *p*-hydroxybenzoate degradation is inhibited if an additional aromatic carbon source is present in the medium (Brzostowicz et al. 2003). In particular, the β-galactosidase activity of the fusion *pobA::lacZ* was negligible when quinate was the carbon source, suggesting that *pobA* expression was repressed (Brzostowicz et al. 2003; DiMarco et al. 1993). Here, *pobA* is up-regulated 29-fold, suggesting that the low β-galactosidase activity observed by Brzostowicz et al. may be caused by a post-transcriptional event (RNA degradation, etc.) rather than the inhibition of *pobA* transcription per se.

#### Genes next to IclR-type regulators were up-regulated during growth on quinate

The binding, at least partially, of PcaU (that belongs to the IclR family) on the binding site of other IclR proteins on the genome may explain why some genes were up-regulated. Six other IclR-type proteins were identified by Gerischer et al. in ADP1 (Gerischer et al. 2008). Their function remains unknown but as in many cases the target of a transcriptional regulator is very close to the regulator gene, we inspected the transcription status of their neighbor genes. Results reported in Table 1 indicated that for 5 out of the 6 genes encoding IclR-type proteins, their neighboring genes were indeed up-regulated. Out of them, the cluster ACIAD0349-0351 is composed of genes highly conserved in proteobacteria. ACIAD0350 contains a domain of unknown function (DUF1656) also present in a cluster of three genes in *E. coli* that form an efflux pump (named AaeXAB). It has been proposed that AaeXAB excretes *p*-hydroxybenzoate when its intracellular concentration is abnormally high (Van Dyk et al. 2004). Members of the same DUF can have different enzymatic reactions but be highly related in their catalytic mechanism (Bastard et al. 2013), thus it cannot be ruled out that AaeXAB and the cluster ACIAD0349-0351 are related in their function. On the other hand, Gerischer et al. also identified 20 putative IclR regulator binding sites in the genome, with 6 located in intergenic regions, making them the most likely candidates for regulator binding (Gerischer et al. 2008). Out of them, 4 were located in the intergenic regions in between *pcaU*-*pcaI* on one side, and *pobR*-*pobA* on the other side. But regarding the 2 remaining putative sites, one is located next to a cluster of 5 genes involved in the synthesis of PQQ (ACIAD2503-2507), which are essential for growth on quinate (de Berardinis et al. 2008). Gerischer et al. speculated that the *pqq* cluster could be, at least partially, influenced by the binding of PcaU. RNA-seq data showed that 4 out of the 5 PQQ genes were up-regulated (Table 1). The last putative binding site is located between *vanK* and *vanA/B* (Table 1). On the one hand, it was suggested that *vanK*, whose product has overlapping specificities with PcaK, is also induced by protocatechuate in ADP1 (D’Argenio et al. 1999). The authors also pointed out that this metabolite may also induce expression of *vanA* and *vanB*. On the other hand, it was speculated that a IclR protein targets this binding site and effects the expression of *vanK,P* and/or *vanA,B* (Gerischer et al. 2008). Data presented here conciliate these two hypotheses and suggest that ACIAD0985 (pcaU-like) is involved in the transcription of *vanAB*.

**Table 1:** ADP1 genes putatively regulated by IClR-type regulators

ADP1 gene	Description	Neighboring genes	Up-regulation (fold)
ACIAD0347	Putative transcriptional regulator	ACIAD0349 (CHP) ACIAD0350 (CHP) ACIAD0351(CHP)	×34 ×10 ×13
ACIAD1684 (*dcaS*)	Putative transcriptional regulator	ACIAD1687 (*caiB*)	×4
ACIAD1688 (*dcaR*)	Putative transcriptional regulator	ACIAD1689 (*dcaF*) ACIAD1690 (*dcaH*) ACIAD1691 (*dcaC*)	×4 ×3 ×4
ACIAD1822	Putative transcriptional regulator	ACIAD1827 ACIAD1828 ACIAD1829 ACIAD1830	×3 ×4 ×3 ×4
ACIAD0985 (*pcaU*-like)	Putative transcriptional regulator	ACIAD0979 (*vanB*) ACIAD0980 (*vanA*) ACIAD0984 (*salA*-like)	×4 ×4 ×4
Intergenic sequences in ADP1 genome with similarity to PcaU/PobR binding sites	ATG distance	Neighboring genes	Up-regulation (fold)
Start:245976 end:2459992	94 (*pqqA*)	ACIAD2503 (*pqqA*) ACIAD2504 (*pqqB*) ACIAD2505 (*pqqC*) ACIAD2506 (*pqqD*) ACIAD2507 (*pqqE*)	×2 ×5 ×4 ×3 ×4
Start:968161 end:968177	206 (*vanK*)	ACIAD0982 (*vanK*) ACIAD0983 (*vanP*)	×28 ×30

#### Regulation of genes of the catechol branch of the β-ketoadipate pathway

The expression of the genes required for catechol and protocatechuate degradation, respectively, is regulated independently (Fischer et al. 2008), cis,cis-muconate being the major inducer of the catechol branch (Bundy et al. 2002) (Brzostowicz et al. 2003). Thus, the genes involved in the degradation of substrates feeding into the catechol branch were not expected to be up-regulated in cells grown with quinate as the sole carbon source. Surprisingly, many genes were indeed up-regulated. This was the case for the *areCBA* genes, which are necessary for growth on benzyl alkanoates (Fig. 1; Online Resource 2, Table S-4). *areCBA* was suggested to be an operon induced by aromatic compounds as benzyl acetate, benzyl alcohol, and benzaldehyde (Jones and Williams 2001). Next, the *salED* genes, located close to the *areCBA* operon (Online Resource 1, Figure S-2B), were also up-regulated (Fig. 1; Online Resource 2, Table S-4). *salED* and *salAR*, which metabolize salicylate and salicylate esters, were proposed to be organized into two convergent transcription units in ADP1 (Jones et al. 2000). The authors hypothesized that *salDE* could be cotranscribed with *areCBA*. Consistent with this hypothesis, RNA-seq data showed that *salED* and *areCBA* were up-regulated while the transcription of *salAR* remained unchanged. Nevertheless, the up-regulation for these genes has never been reported in the absence of the substrates of their encoded enzymes. Finally, in the same catabolic island the *cat* genes involved in catechol degradation are organized in two transcriptional units: *catA* on the one hand and the *catBCIJFD* operon on the other hand (Romero-Arroyo et al. 1995). Here, *catA* was up-regulated (Fig. 1; Online Resource 2, Table S-4), despite the absence in the culture medium of any aromatic compound that could be degraded into the inducer cis, cis-muconate.

### Metabolomics

#### Selection of the chromatographic conditions

To analyze the metabolome of ADP1, we aimed to set up an efficient liquid chromatography method for the separation of the metabolites prior to their detection by high-resolution mass spectrometry. The chromatographic separation step is critical to limit ion suppression effects (King et al. 2000; Taylor 2005). We focused on the detection of the intermediates of the central carbon metabolism, i.e. polar metabolites, and on the quantification of AMP, ADP, and ATP, to estimate the energy charge of the cell (see below). A first chromatographic method took advantage of the widely used C18-UHPLC technology. Alternatively, the more recent ZIC-*p*HILIC column was evaluated, as hydrophilic interaction liquid chromatography has recently been successfully used in metabolomic studies (Kamleh et al. 2008; Nguyen and Schug 2008; t’Kindt et al. 2010). AMP, ADP and ATP eluted from the ZIC-*p*HILIC column at ~11, 13 and 15 min, respectively, with an asymmetry factor close to 1 (not shown), allowing quantification in biological samples. It is worthy to note that such values for these 3 nucleotides could only be obtained with a pH value of 9.9 for the aqueous mobile phase. On the opposite, with the C18 column, AMP, ADP and ATP were poorly retained (<2 min) with an asymmetry factor >2, impeding quantification. Such a lack of retention on a C18 column has been previously reported (Coulier et al. 2006). To further compare the retention and separation properties of the two chromatographic columns, 121 commercial compounds representative of the diversity of the metabolism were analyzed in both positive and negative ionization modes (Online Resource 1, Table S-5). These reference metabolites ranged from *m*/*z* values of 68 to 809, and comprised amino acids, amino acids precursors or derivatives, nucleosides, nucleotides, organic acids, vitamins and derivatives, and redox electron carriers. Eight of these compounds could not be detected on at least one column, but more than 95 % were retained on the ZIC-*p*HILIC column and only ~20 % on the C18 column (Online Resource 1, Figure S-3). In conclusion, all metabolomics investigations were conducted with the ZIC-*p*HILIC column.

#### Reproducibility of metabolomics sampling

We intended to investigate the metabolism of exponentially growing cells of ADP1 through representative metabolome preparations, in which the metabolome composition has to be as close as possible to the metabolite content of the cultured cells at the moment of sampling. As metabolites can have a lifetime of seconds and even less for ATP (Walsh and Koshland 1984), this required rapid cell quenching combined with cell inactivation to ‘freeze’ the microbial metabolism. To evaluate the quality of metabolism quenching, along with the extraction and analytical methods employed, we calculated the adenylate energy charge (AEC) i.e. the amount of metabolically available energy of the cells, according to the formula proposed by (Chapman et al. 1971),

$${\text{AEC }} = \;\frac{{\left[ {ATP} \right] + 0.5[ADP]}}{{\left( {\left[ {ATP} \right] + \left[ {ADP} \right] + \left[ {AMP} \right]} \right)}}.$$

The AEC values obtained from the same carbon source were highly reproducible. A value of 0.84 ± 0.01 was obtained for cells grown on succinate, which is consistent with the one proposed by Chapman et al. for cells of *E. coli* during normal growth (Chapman et al. 1971). Surprisingly, quinate-grown cells displayed a lower value of 0.66 ± 0.08. The reproducibility of this value indicated that it was probably not related with an incorrect handling of the cells during sampling and LC/MS analysis, but rather a characteristic of growth on quinate. It is also ruled out that the cells entered a starvation phase during their cultivation. Studies have shown that the level of global negative supercoiling of chromosomal DNA is controlled by the cellular energy charge (Higgins et al. 1988; Hsieh et al. 1991). A variation of the AEC, in response of environmental conditions, can trigger a change of DNA superhelicity, affecting the entire chromosome and the expression levels of all operons whose promoters are sensitive to superhelicity. Hatfield and Benham proposed that, in this way, the global pattern of gene expression may be dynamically tuned to changing needs of the cell under a wide variety of circumstances (Hatfield and Benham 2002). Thus, the global transcriptional response elicited by quinate in ADP1 may be linked to the energy charge instead of the growth rate. Alternatively, it cannot be excluded that quinate is a substrate that does not obey the hypothesis that gene expression depends directly on bacterial growth (Klumpp et al. 2009).

#### Global analysis of the metabolome

##### Data processing

XCMS automatic peak detection yielded 1,258 peaks in positive ionization mode and 1,574 in negative ionization mode, classified in 227 and 356 feature groups, respectively. Data were further examined to remove non-relevant peaks and incorrect data-gathering, leading to a final list of 451 validated metabolites: 280 in positive ionization mode, 171 in negative ionization mode, and 38 in both ionization modes (Online Resource 2, Table S-6). 352 could be matched to known compounds querying metabolic databases (Kegg and/or Metlin) and 99 remained without any proposition. The retention time, accurate mass, and CID spectrum profile when available (low mass cut-off of fragmentations or low signal intensity being limiting factors) of the putatively identified metabolites were compared to those of commercial standards. Finally, 105 metabolites could be identified with high confidence according to the Metabolomics Standards Initiative (Sumner et al. 2007) (Online Resource 2, Table S-7). The quality of data processing by XCMS is illustrated in Online Resource 1, Figure S-4 for one metabolite representative of run to run retention time drift (alanine; M90T682) and in Figure S-5 for one metabolite characteristic of a low abundant species (cytosine; M112T459). We used a PCA to visualize the structuring of our final data set, and found that all samples corresponding to the same growth condition grouped together (Online Resource 1, Figure S-6), illustrating the robustness of our data. The clear separation between the 2 groups showed that the nutritional regime is the main parameter differentiating the metabolomes. The reproducibility of our sample preparation was further confirmed by the RSD values calculated from biological replicates (<40 %) for each carbon source, all being consistent with published data (Bajad et al. 2006; Fiehn et al. 2000; van der Werf et al. 2008).

##### Metabolome overview

**Fig. 3:**
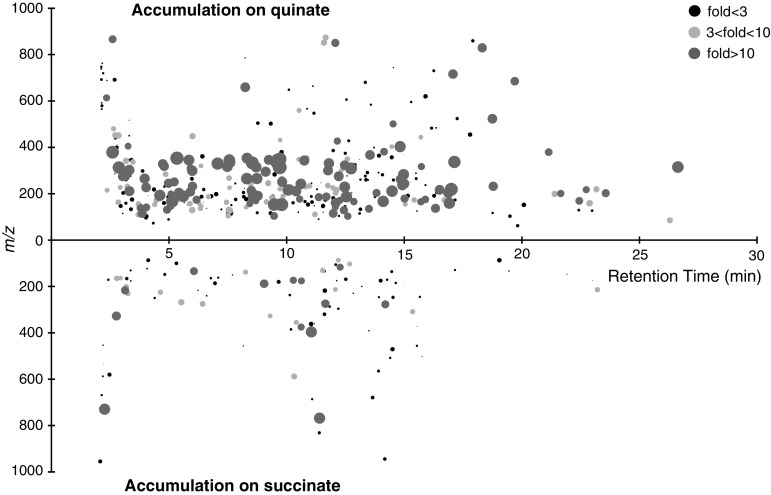
Visualization of the 451 LC/MS-detected metabolites of *A. baylyi* ADP1. The retention time on the ZIC-*p*HILIC column is represented by position on the *x*-axis. Mass-to-charge ratio is represented by position on the *y*-axis. Fold change is indicated by* color and radius* of each metabolite (log scale). *Upper panels* metabolites accumulating in quinate-grown cells. *Lower panels* metabolites accumulating in succinate-grown cells

The concerted effects of changes in gene expression due to changes in the environment, observed through RNA-seq data, should be ultimately reflected in the metabolome. This was actually the case, as Fig. 3 shows that the metabolome was dramatically affected. Metabolites eluted along the whole chromatographic gradient and ranged from *m*/*z* 62 to 955, illustrating their great chemical and structural diversities. They were more concentrated in quinate-grown cells, with numerous molecules accumulating more than tenfold. 227 metabolites had their concentration modified, indicating that ~50 % of LC/MS-detected metabolites were affected by the carbon source. For those whose concentration was most affected and which were more abundant in quinate-grown cells, some could not be matched to metabolic databases. For the ones that matched to databases, most of the proposed identities did not correspond to expected metabolites for ADP1 and have therefore to be further verified experimentally. A first hypothesis could be that for some of them, identities are indeed correct, these metabolites being unpredicted in ADP1. Alternatively, proposed identities could be wrong because the structure of some of these metabolites has not been reported so far. In both cases, these metabolites would represent an unknown side of ADP1 metabolism. Determining their identity would thus be a priority.

**Fig. 4:**
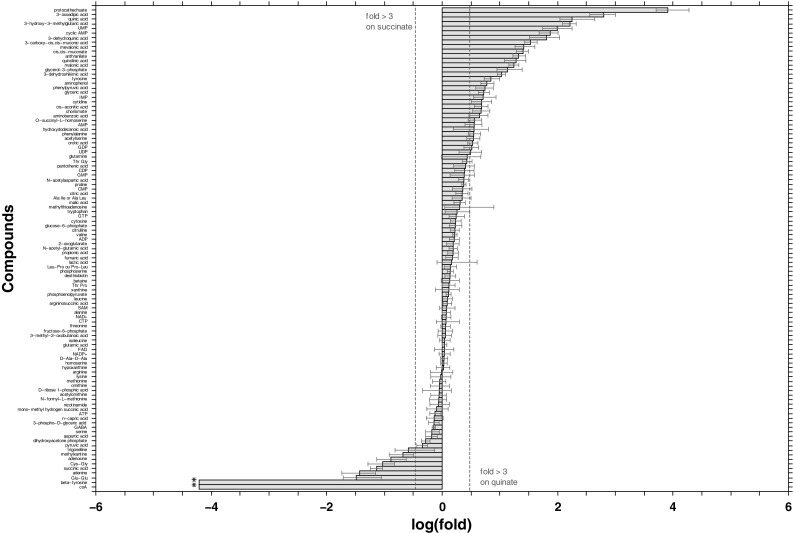
Differences for identified metabolites in ADP1 metabolomes. Alterations are expressed as log(fold). The confidence intervals of log(fold) at 95 % (*red whiskers*) were determined using Fieller’s formula (Fieller 1954) derived from the *t* test for the ratio of two means with unequal variances (Tamhane and Logan 2004). Data represent the average for 6 independent succinate metabolomes and 4 quinate metabolomes.* Dotted blue lines* correspond to a fold-change of 3. *Asterisk* indicates metabolites for which the fold-change is infinite (absence in quinate-grown cells) (Color figure online)

Concerning the 105 identified metabolites, their distribution pattern, presented in Fig. 4, also shows that more metabolites accumulated during growth on quinate. More than 70 belonged to metabolic pathways related to central metabolism (metabolism of amino acids, nucleotides, and TCA cycle), and for most of them, their concentration was not affected by the carbon source. This suggests that the central metabolism of ADP1 was independent of the nature of the carbon source, which is in agreement with reports on related organisms (Frimmersdorf et al. 2010; van der Werf et al. 2008). Nevertheless, out of these 105 metabolites identified, 41 had their concentration affected by the carbon source shift. These included the expected catabolic intermediates of quinate degradation that accumulated in quinate-grown cells, as protocatechuate, 3-oxoadipate, dehydroquinate, and dehydroshikimate. 3-Carboxy-cis, cis-muconate acid was also detected, its fragmentation pattern being consistent with its structure (Online Resource 1, Figure S-7). These results agreed with RNA-seq data showing that all the known genes involved in quinate degradation were highly up-regulated. But metabolites not related to quinate catabolism had their concentration affected as well. This was the case for compounds belonging to the metabolism of pyrines and pyrimidines as UDP, GDP, orotate, cytidine, and IMP (3 ≤ fold change ≤ 5) or adenosine, adenine, and UMP (7 ≤ fold change ≤ 120).

##### Aromatic amino acid metabolism is affected in quinate-grown cells

Out of the 17 proteinogenic amino acids identified, only 2 had their concentration significantly changed and accumulated on quinate: the aromatic amino acids phenylalanine (threefold) and tyrosine (sevenfold). Different identified precursors for the biosynthesis of the aromatic amino acids were also more abundant in quinate-grown cells, as chorismate (fourfold), anthranilate (21-fold), and phenylpyruvate (fivefold). Aromatic amino acids are synthesized from phosphoenolpyruvate and d-erythrose 4-phosphate via cytosolic anabolic 3-dehydroquinate and 3-dehydroshikimate. A first hypothesis is that the genes involved in the biosynthesis of these amino acids were up-regulated in quinate-grown cells. However, their transcription was not significantly modified. Alternatively, the accumulation of the two aromatic amino acids and their precursors could be caused by a leak, from periplasm to cytoplasm of metabolites that are common to both quinate degradation and shikimate synthesis, such as 3-dehydroquinate and/or 3-dehydroshikimate. This unsuspected transport, which could involve VanK and/or PcaK would in turn enhance the biosynthetic flux of aromatic amino acids, leading to their accumulation in the cell. ACIAD3353, coding for 3-dehydroquinate synthase (EC 4.2.3.4) and ACIAD1738, coding for 3-dehydroquinate dehydratase (EC 4.2.1.10) are obligatory genes involved in the synthesis of the anabolic 3-dehydroquinate and 3-dehydroshikimate. The deletion of either ACIAD3353 or ACIAD1738 is lethal for cells grown on succinate (de Berardinis et al. 2008). According to our alternative hypothesis, their deletion should not impede cell growth if quinate is the carbon source, the disruption of the normal biosynthetic pathway being complemented by transport in the cytoplasm of catabolic 3-dehydroquinate and 3-dehydroshikimate for aromatic amino acid synthesis. Hence, the 2 deletion mutants (ΔACIAD3353 and ΔACIAD1738) were constructed. Clones for each mutant were first selected on plates of MA medium containing succinate as the carbon source, and supplemented with kanamycin to select Kan^R^ colonies and shikimate to allow aromatic amino acid biosynthesis. For both mutants, succinate-grown cells were auxotroph for shikimate while quinate-grown ones remained prototroph (Online Resource 1, Table S-8). These results indicate that when quinate is metabolized, a flux of 3-dehydroshikimate crosses the cytoplasmic membrane and feeds into the aromatic amino acid pathway. Further studies would be required to explain the unaffected concentration of tryptophan.

##### Identified metabolites were not predicted by ADP1 genome-scale metabolic model

We previously reported on the construction of a constraint-based metabolic model of ADP1, iAbaylyiv4 (Durot et al. 2008). Genome-scale metabolic models are powerful tools to study global properties of metabolic networks. This model allowed generating in silico metabolites, according to the carbon source used (not shown). We observed that some of the metabolites experimentally identified in the metabolomes of ADP1 were actually not predicted by the model for quinate- and/or succinate-grown cells. For example, γ-aminobutyrate (GABA, Online Resource 2, Table S-7 and Online Resource 1, Figure S-8) is not proposed by iAbaylyiv4: while *gabT* (ACIAD3446*)* and *gabD* (ACIAD3445), involved in the conversion of GABA to succinate are identified, the annotation of a gene encoding a GABA producing glutamate decarboxylase is missing. It was previously observed that the genes coding for the E1 and E2 components of the 2-oxoglutarate dehydrogenase complex, *sucA* (ACIAD2876) and *sucB* (ACIAD2875), were not essential (de Berardinis et al. 2008), although their deletion, disrupting the TCA cycle, should be lethal. The identification of GABA in ADP1 metabolome supports the hypothesis that a non-identified glutamate decarboxylase helps in succinate formation from 2-oxoglutarate through the GABA shunt. Malonate (Online Resource 1, Figure S-9) and 3-hydroxy-3-methylglutarate (Online Resource 1, Figure S-10) could be the hydrolysis products of the corresponding Coenzyme A esters in the chromatographic mobile phase, as these compounds are unstable at alkaline pH (Joyard and Stumpf 1980). However, these compounds gave clearly defined peaks (not shown) while hydrolysis products would give tailing peaks since it would not be instantaneous. While malonyl-CoA is an obligate intermediate in fatty acids synthesis, 3-hydroxy-3-methylglutaryl-CoA is not expected, but ADP1 contains a putative hydroxymethylglutaryl-CoA lyase (ACIAD2820) that could metabolize it into 3-hydroxy-3-methylglutarate. 3-hydroxy-3-methylglutaryl-CoA also belongs to the mevalonate pathway for terpenoid biosynthesis, which is not either predicted to occur in ADP1 (Barbe et al. 2004). Nevertheless, it is noteworthy that mevalonate was also potentially identified in the metabolome (Online Resource 2, Table S-7 and Online Resource 1, Figure S-11) and that 3-hydroxy-3-methylglutarate and mevalonate both accumulated in quinate-growth conditions (164-fold and 26-fold, respectively), suggesting that they could be related. *N*-acetylaspartate, mono-methyl hydrogen succinate, and trigonelline were also identified (Online Resource 2, Table S-7 and Online Resource 1, Figures S-12, 13, 14) although, to our knowledge, these compounds have never been reported to occur in bacteria so far. We could putatively identify cis–cis muconate comparing its fragmentation pattern to the one of its counterpart in the protocatechuate branch, 3-carboxy-cis,cis-muconate. This compound, known only as a catabolite of the catechol branch was not expected either (Online Resource 1, Figure S-7).

Finally, ~15 % (16/105) of the metabolites experimentally identified in ADP1 metabolome were not predicted by iAbaylyiv4 model. Although there is continuous progress on integrating omics analyses in metabolic models, the modeling of metabolic networks remains simplified and limited (Oh et al. 2007). This is mostly due to the fact that many reactions that play part in metabolism have not been studied. At the present time, the annotation of the ADP1 genome is incomplete, and ∼30 % of the genes are still annotated as ORFs of unknown function. Therefore, our genome-scale model obviously lack metabolic capabilities that ADP1 possesses.

To conclude, 28 unidentified compounds were present exclusively in quinate-grown cells (Online Resource 2, Table S-6). None of them could be identified and moreover, for 18 of them no putative identity could be assigned by Kegg database. Thus, data suggest that during the global transcriptional response triggered by quinate, unsuspected enzymatic activities took place and led to the formation of unknown metabolites that are not related to the current knowledge of the quinate dissimilation pathway. In other words, this study, that integrated transcriptomic and metabolomic approaches, gave indication that all the enzymatic reactions and metabolic pathways in ADP1 have not yet been identified.

### Growth phenotyping of ADP1 collection of deletion mutants

To identify the genes participating in the catabolism of quinate, our laboratory previously conducted the profiling for growth on quinate of the whole collection of deletion mutants (de Berardinis et al. 2008). Nevertheless, the essentiality of all the expected genes involved in quinate degradation could not be determined due to their lack at the time of the growth phenotyping (de Berardinis et al. 2008). Here, data for these mutants were incorporated to those previously published (Online Resource 2, Table S-9). All the genes coding for the enzymes involved in the transformation of quinate to succinyl-CoA and acetyl-CoA are essential, with the exception of *pcaD* and *pcaF* (Fig. 1 and Online Resource 20). It was proposed that the dispensability of *pcaD* could be explained by the presence of the isofunctional gene *catD* (de Berardinis et al. 2008) and similarly, *pcaF* could be complemented by *catF*. However, RNA-seq data indicated that although *pcaD* and *pcaF* were both highly up-regulated (117- and 443-fold respectively, Online Resource 2, Table S-4), *catD* and *catF* were not up-regulated and only weakly transcribed (52 and 41 reads, respectively). These low expression levels may be not sufficient for substantial CatD and CatF activities and may suggest that other enzymes with overlapping substrate specificity were responsible for the rescue of the *pcaD* and *pcaF* deletions. For example, the degradation of dicarboxylic acids converges with the β-ketoadipate pathway at the level of β-ketoadipyl-CoA (Fig. 1). RNA-seq data indicated that the expression of *dcaF*, which is isofunctional with *pcaF* and *catF*, was induced on quinate and may rescue *pcaF* deletion. Alternatively, an increase in transcription of *catD* and *catF* in the corresponding mutants ∆ACIAD1708 (Δ*pcaD*) and ∆ACIAD1706 (Δ*pcaF*) could explain the dispensability of *pcaDF*. Further transcriptional analysis conducted in these mutants is required to confirm of this hypothesis. However, some mutants that show a growth defect could not be easily linked to the current knowledge of quinate metabolism and would thus require more investigation. For example, most of the genes of the *nuo* operon (*nuoAEFGHIJKMN*), coding for NADH dehydrogenase I, the proton-pumping NADH:ubiquinone oxidoreductase (respiratory complex I) that couples the electron transfer from NADH to ubiquinone with the translocation of protons across the membrane, were essential for growth on quinate (de Berardinis et al. 2008). This enzymatic complex was not required for growth on succinate, as succinate dehydrogenase (respiratory complex II) is a second entry point to the electron transport chain. But NADH dehydrogenase I was not either anticipated to be essential for growth on quinate since purified quinate dehydrogenase from *Acinetobacter* *calcoaceticus* transferred electrons from PQQ directly to ubiquinone (Adachi et al. 2003). More generally, PQQ-dependent membrane-bound dehydrogenases are supposed to feed electrons directly to ubiquinone in the respiratory chain and thus bypass the need for respiratory complex I (Yamada et al. 2003). Nevertheless, growth profiling results suggest that quinate dehydrogenase interacts with NADH I dehydrogenase for electron transfer. Likewise, it is surprising that genes that code for enzymes such as *panD* (ACIAD2911), *glnE* (ACIAD0596) and *alkK* (ACIAD1818), transport proteins (ACIAD1590 and 1601) or proteins of unknown function (ACIAD3137, 2741, 2176, and 2066) were also necessary for growth on quinate. The growth of all these mutants was not affected on other carbon sources such as acetate or glucose (de Berardinis et al. 2008), ruling out the possibility of a bias in their genetic construction and/or selection. This suggests that these genes are indeed related in some way to quinate catabolism.

## Concluding remarks

The analysis of the *ADP1* transcriptome and metabolome confirmed that quinate triggered a specific transcriptional and metabolic response that allowed its dissimilation. But it essentially revealed that quinate elicited a global transcriptional response that affected 12 % of the total number of the genes, most of them being of unknown function. We suggest that this global response, that was independent of the growth rate of the bacterium, was instead connected to its energy charge. These important changes in gene expression were reflected in the metabolome. Although metabolites of the central metabolism were not affected, the concentration of the majority of metabolites was indeed modified. Structural elucidation of some metabolites that were produced exclusively in quinate-grown cells is currently ongoing. It will help to find their associated genes and shed light on putative hidden metabolic pathways.

## Electronic supplementary material

Below is the link to the electronic supplementary material.

Supplementary material 1 (PDF 1276 kb)

Supplementary material 2 (XLSX 1019 kb)
