# Supplementary material for: Novel metabolic features in Acinetobacter baylyi ADP1 revealed by a multiomics approach
Source: Metabolomics. 2014 Apr 29;10(6):1223–38. doi: 10.1007/s11306-014-0662-x (PMC4213383; doi:10.1007/s11306-014-0662-x)
Supplement: Supplementary file 1 — Supplementary material 1 (PDF 1276 kb) [file 11306_2014_662_MOESM1_ESM.pdf]

Supplementary materials for manuscript

NOVEL METABOLIC FEATURES IN ACINETOBACTER BAYLYI ADP1 REVEALED BY A MULTIOMICS APPROACH

[1-3]Lucille Stuani, [1-3]Christophe Lechaplais, [1-4]Aaro V. Salminen, [1-3]Béatrice Ségurens, [1-3]Maxime Durot, [1-3]Vanina Castelli, [1-3]Agnès Pinet, [1-3]Karine Labadie, [1-3]Stéphane Cruveiller, [1-3]Jean Weissenbach, [1-3]Véronique de Berardinis, [1-3]Marcel Salanoubat, [1-3]Alain Perret*

[1]Direction des Sciences du Vivant, Commissariat à l'Energie Atomique et aux Energies Alternatives (CEA), Institut de Génomique, Evry, France.
[2]CNRS-UMR8030, Evry, France.
[3]Université d'Evry Val d'Essonne, Evry, France.
[4]Tampere University of Technology, Department of Chemistry and Bioengineering, Korkeakoulunkatu 10, 33720 Tampere, Finland

*aperret@genoscope.cns.fr; Tel: 33-1-60-87-45-90; Fax: 33-1-60-87-25-14

Check list

Protocol S-1 Dehydroquinate synthesis

Table S-1 Primers used for the construction of the deletion mutants

Table S-2 AMP, ADP and ATP calibration curves in positive ionization mode with AEC calculations using RAW and XCMS data

Table S-3 Summary of the RNA sequencing coverage data

Table S-4 Expression profiles of log phase cultures of *Acinetobacter baylyi* ADP1 grown on succinate and quinate

Table S-5 List and details about the 114 commercial metabolites used as standards

Table S-6 Full set of metabolites detected in Acinetobacter baylyi ADP1 metabolome

Table S-7 Identified metabolites in Acinetobacter baylyi ADP1 metabolome

Table S-8 Growth of ADP1 wild type and mutant cells on different media

Table S-9 Acinetobacter baylyi ADP1 mutant growth yields on quinate

Figure S-1 Metabolomic experimental design

Figure S-2 Chromosomal organization of genes involved in the metabolism of aromatic compounds

Figure S-3 Retention times for 114 detected commercial compounds representative of the bacterial metabolism on the C18 and ZIC-pHILIC column

Figure S-4 Quality of XCMS data processing (peak detection, retention time alignment and fold-change) for alanine (M90T682)

Figure S-5 Quality of XCMS data processing (peak detection, retention time alignment and fold-change) for cytosine (M112T459)

Figure S-6 PCA scores plot of the effect of the carbon source change on the metabolome of Acinetobacter baylyi ADP1

Figure S-7 Extracted ion chromatograms (XIC) and tandem mass spectra for putative 3-carboxy-cis,cis-muconic acid and cis,cis-muconic acid in negative ionization mode in biological samples

Figure S-8 Tandem mass spectrum for GABA in negative ionization mode and positive ionization mode

Figure S-9 Tandem mass spectrum for malonic acid in negative ionization mode

Figure S-10 Tandem mass spectrum for 3-hydroxy-3-methylglutaric acid in negative ionization mode

Figure S-11 Tandem mass spectrum for mevalonic acid in negative ionization mode

Figure S-12 Tandem mass spectrum for N-acetyl aspartic acid in negative ionization mode

Figure S-13 Tandem mass spectrum for mono-methyl hydrogen succinic acid in negative ionization mode

Figure S-14 Tandem mass spectrum for trigonelline in positive ionization mode

Protocol S-1 Dehydroquinate synthesis

ADP1 was grown in 150 ml of quinate-containing growth medium to induce quinate dehydrogenase production. Cells were harvested after centrifugation (30 min, 2500 g, 4°C). The cell pellet was suspended in 1 ml of 50 mM phosphate buffer; pH 8.0, containing 1 µl Lysonase (Novagen) and 10% (v/v) BugBuster (Novagen) and let for 20 min at room temperature under vigorous shaking, and centrifuged (30 min, 20 000 g, 4°C). The supernatant was recovered and assayed for quinate dehydrogenase activity according to Adachi *et al*. (Adachi et al. 2003). For the use of enzymatically produced dehydroquinate as a reference compound for metabolite identification, 11 µg of lysate proteins were incubated in 1 ml of 50 mM phosphate buffer; pH 8.0, containing 10 mM quinate for 60 min. Proteins were discarded using 3K centrifugal PES filters, and 200 µl of the mixture were added to 800 µl 80/20 acetonitrile/ammonium carbonate pH 9.9 for LC/MS analysis.

REFERENCE:

Adachi, O., et al. (2003). Purification and characterization of membrane-bound quinoprotein quinate dehydrogenase. Biosci Biotechnol Biochem 67, 2115-23

Table S-1 Primers used for the construction of the deletion mutants

Primers	Sequences
Primers for the integration cassette	
Primer_1:	5'-CCCAGCCTCCAATTCAAATCATAAAAAAT-3'
Primer_2:	5'-CCAGCTCCGCATGCTTAGAAAAACTCATCG-3'
Primers for ΔACIAD1738	
Primer_3:	5'-TCGCTATAAATAATCGCTACGC-3'
Primer_4:	5'-TTTTTATGATTTGAATTGGAGGCTGGGTCAACGTGCTCCAATTTC-3'
Primer_5:	5'-CGATGAGTTTTTCTAAGCATGCGGAGCTGGTTCAACATGATTTAGGAACATTC-3'
Primer_6:	5'-TAATACCCAATGTTTCACCAGC-3'
Primer_7:	5'-ATCAAATTGGTCAGTCCATCAC-3'
primer_8:	5'-GACCAAATTGGATTACATCACC-3'
Primers for ΔACIAD3353	
Primer_3:	5'-TGAGCTTCAGTTACAGCGTACC-3'
Primer_4:	5'-TTTTTATGATTTGAATTGGAGGCTGGGTCTGAGGATTTAGCTGACTACC-3'
Primer_5:	5'-CGATGAGTTTTTCTAAGCATGCGGAGCTGGAAGCAAGTCATACATAAGGTG-3'
Primer_6:	5'-CATCAGAAGTACGAAAAACCTC-3'
Primer_7:	5'-GACGGGATTTAGTGTTAGCAAC-3'
Primer_8:	5'-CGCTGCACTGTCACTCGTAAAG-3'

Table S-3 Summary of the RNA sequencing coverage data

Statistics	Carbon source	
	Succinate	quinate
Total number of reads	25,986,330 ± 5,046,266	29,840,121 ± 5,467,425
Total reads (Gb)	1.97	2.27
Genome coverage (fold)	503	580
Mapped reads (%)	97.46 ± 0.16	97.31 ± 1.13
Unique matches (%)	90.22 ± 0.71	82.40 ± 1.03
Total unmapped reads (%)	2.54 ± 0.16	2.69 ± 1.13
Reads mapped on rRNA (%)	6.06 ± 1.00	13.91 ± 0.40

Table S-5 Names, molecular mass and retention time of the 114 commercial metabolites detected by LC/MS. Each compound is injected at 50 µM. Isoprene, butanal, deoxyribose, histidinol, pyridoxamine-5-phosphate, thyramine, and diaminopropane could not be detected on at least one column.

Metabolite name	Molecular mass	Retention time on C18 (min)	Retention time on ZIC-pHILIC (min)
Propanoate	74.0368	1.22	4.47
Glycine	75.032	0.96	11.28
Pyruvate	88.016	1.02	4.24
Butanoic acid	88.0524	1.83	3.58
Alanine	89.04767	0.96	10.63
Lactate	90.0317	0.99	5.16
Acetoacetate	102.0317	1.06	10.77
Gamma-Aminobutyrate (GABA)	103.0633	0.97	11.17
Hydroxypyruvate	104.011	0.9	6.89
Serine	105.0426	0.94	11.3
D-Glyceric acid	106.0266	0.94	7.51
Catechol	110.0368	4.6	3.78
Isocytosine	111.0433	1.38	7.2
Cytosine	111.0433	1.18	7.74
Uracil	112.0273	1.35	5.36
Proline	115.0633	1.07	9.1
Fumarate	116.011	0.9	11.16
3-Methyl-2-Oxobutyrate	116.0473	1.4	7.75
Betaine	117.07897	1	7.8
Valine	117.079	1.21	8.91
Succinate	118.0266	1.12	10.5
Threonine	119.0582	0.99	10.8
Homoserine	119.0582	0.96	10.78
Mercaptopyruvate	119.9881	1.07	11.54
Cysteine	121.0197	1.17	11.94
Nicotinamide (Niacinamide)	122.048	2.66	4.36
Nicotinate	123.032	1.74	3.87
Taurine	125.0147	0.95	10.31
Thymine	126.0429	2.62	4.31
4-Methyloxovalerate	130.0629	2.63	2.72
2-Ketohexanoate	130.06299	2.67	2.69
(Iso)Leucine	131.0946	1.8	7.95
Oxaloacetate	132.0059	0.91	14.15
Ornithine	132.08987	0.91	18.84
Aspartate	133.0375	0.92	10.35
Malate	134.0215	0.92	11.19
Adenine	135.0545	1.67	6.27
Hypoxanthine	136.0385	1.62	6.77
p-Aminobenzoate	137.0477	1.72	6.19
Anthranilic acid	137.0477	3.13	8.24
Trigonelline	137.0477	1.05	8.23
2-Ketoglutarate	146.0215	0.97	16.44
Glutamine	146.0691	1.35	10.68

Lysine	146.1055	0.92	20.54
Glutamate	147.0532	0.93	10.07
Methionine	149.051	1.4	8.08
Guanine	151.0494	1.59	8.67
Xanthine	152.0334	1.6	7.44
2.3-Dihydroxybenzoic acid	154.0266	1.49	4.18
Protocatechuate	154.0266	1.89	8.94
Histidine	155.0695	1.18	12.84
Orotic acid	156.0171	1.04	5.87
Allantoin	158.0439	1.02	9.62
Phenylpyruvate	164.0473	1.64	9.29
7-Methylguanine	165.0651	2.51	6.5
Phenylalanine	165.079	3.82	6.88
Phosphoenolpyruvate	167.9824	0.91	12.62
Pyridoxamine	168.0899	1.91	8.74
Pyridoxine	169.0739	2.02	4.84
n-Capric acid	172.1463	10.92	2.38
Aconitate	174.0164	0.9	12.69
Shikimate	174.0528	0.95	9.47
N-Acetylornithine	174.1004	1.05	11.05
Arginine	174.1117	0.96	22.02
Citrulline	175.0957	1.01	11.52
Glucosamine	179.0794	0.96	11.33
Hydroxyphenylpyruvate	180.0423	1.91	11.41
myo-Inositol	180.0634	0.99	12.28
Tyrosine	181.0739	1.57	9.28
3-Phosphoglycerate	185.9929	0.85	12.24
Citrate	192.027	0.92	13.08
Tryptophan	204.0899	4.52	8.32
Pantothenic acid	219.1107	1.93	4.5
Cystathionine	222.0674	0.95	12.81
Deoxycytidine	227.0906	2.03	7.17
Deoxyuridine	228.0746	2.65	5.06
Thymidine	242.0903	4.16	4.22
Cytidine	243.0855	1.55	8.37
Uridine	244.0695	1.96	6.52
Biotin	244.0882	3.09	4.74
Deoxyadenosine	251.1018	4.5	5.02
Deoxyinosine	252.0859	3.4	6.29
Glucosamine-6-phosphate	259.0457	0.87	11.51
Glucose-6-phosphate	260.0297	0.92	11.91
Thiamine	265.1123	1.49	20.78
Adenosine	267.0968	4.36	5.84
Inosine	268.0808	3.13	7.49
Guanosine	283.0917	3.31	8.96
Xanthosine	284.0757	2.17	7.94
dCMP	307.0569	1.03	10.26
Reduced Glutathione	307.0838	1.01	13.28
CMP	323.0519	0.94	11.08
UMP	324.0359	0.98	10.5
Cyclic AMP	329.0525	4.17	5.38
AMP	347.0631	1.26	9.38
dGMP	347.0631	1.22	10.88
IMP	348.0471	1.15	10.8

GMP	363.058	1.11	11.71
Riboflavin	376.1383	6.31	5.43
S-Adenosyl-L-methionine	398.1372	1.18	12.83
CDP	403.0182	0.85	12.22
ADP	427.0294	1.05	10.77
dGDP	427.0294	1.01	12.07
Folate	441.1397	5.31	11.78
CTP	482.9845	0.84	13.23
ATP	506.9957	0.99	11.84
dGTP	506.9957	0.88	13.08
GTP	522.9907	0.86	13.9
Oxidized Glutathione	612.152	0.98	12.12
NAD	664.1169	1.32	9.96
NADP	744.0833	0.96	12.08
CoA	767.1152	3.78	10.02
FAD	785.1571	4.07	7.81
Acetyl-CoA	809.1258	5.05	8.98

Table S-8 Growth of ADP1 wild type and mutant cells on different media

Strain	Medium used for growth		
	succinate + shikimate	succinate	quinate
Wild type	+	+	+
Δ3353	+	-	+
Δ1738	+	-	+

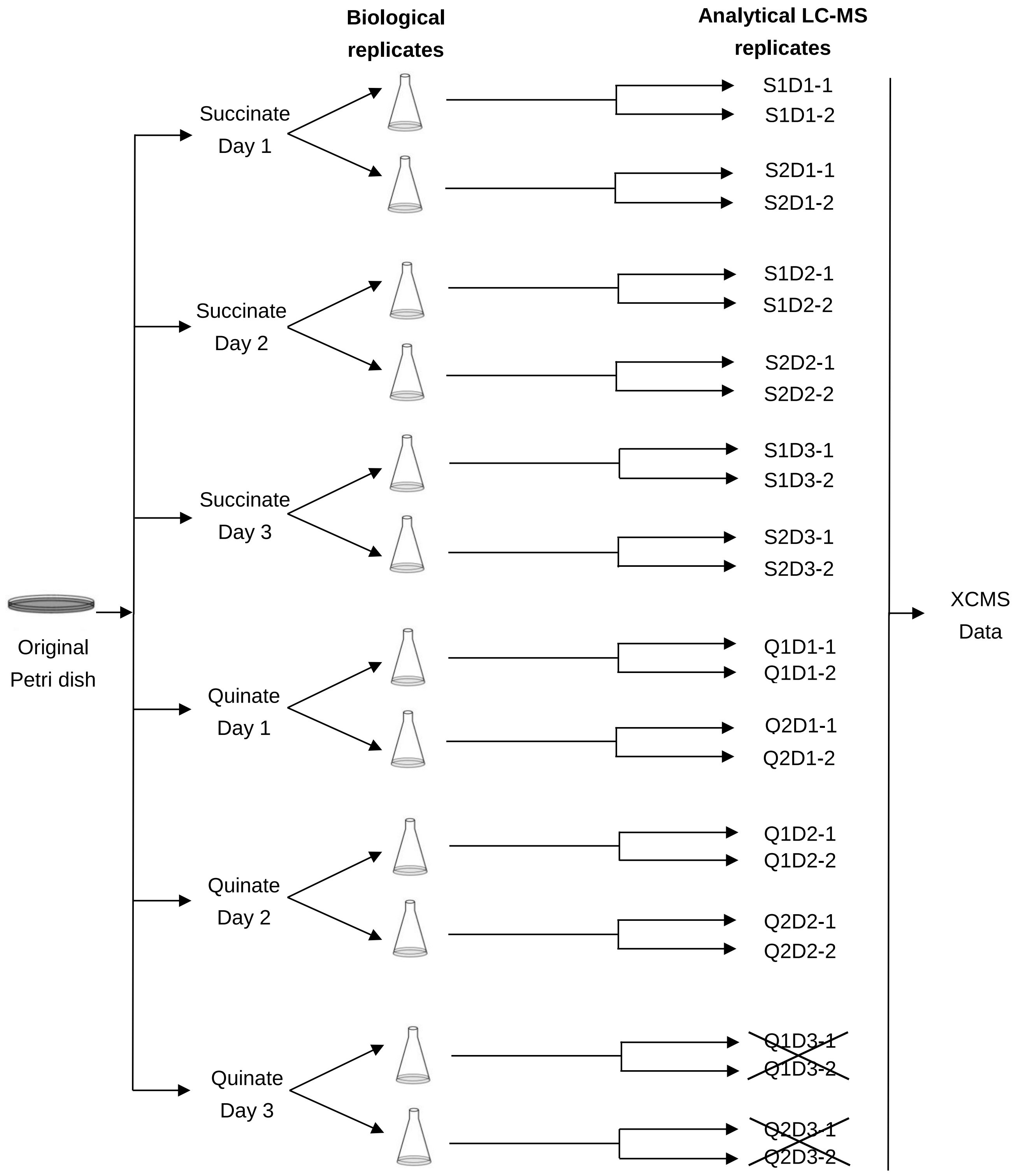

Figure S-1 Metabolomic experimental design - Experiments were conducted during 3 days; **c**ultures were performed in duplicates for each carbon source and the samples were analyzed in duplicate by LC-MS. Samples Q1D3-1, Q1D3-2, Q2D3-1, and Q2D3-2 were not taken into account.

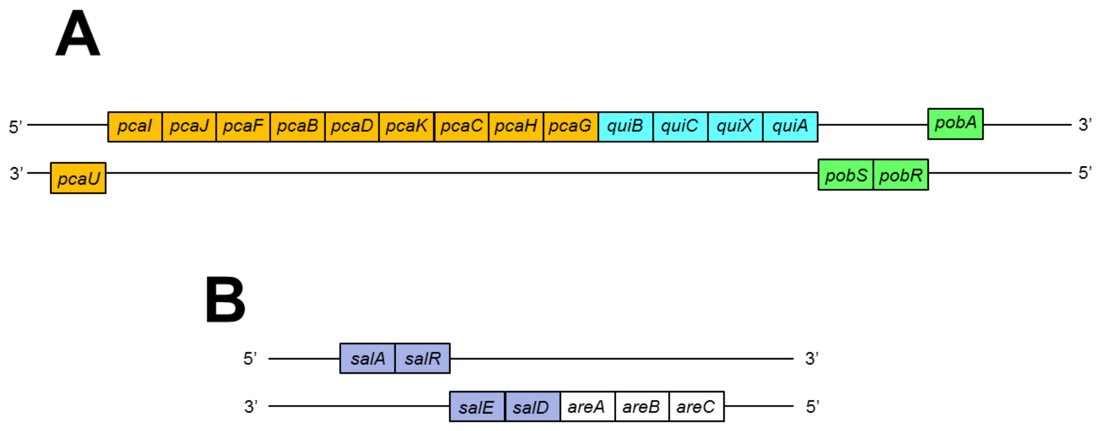

Figure S-2 Chromosomal organization of genes involved in the metabolism of aromatic compounds. A: the *pca-qui-pob* cluster (protocatechuate branch). B: the *sal-are* cluster (catechol branch).

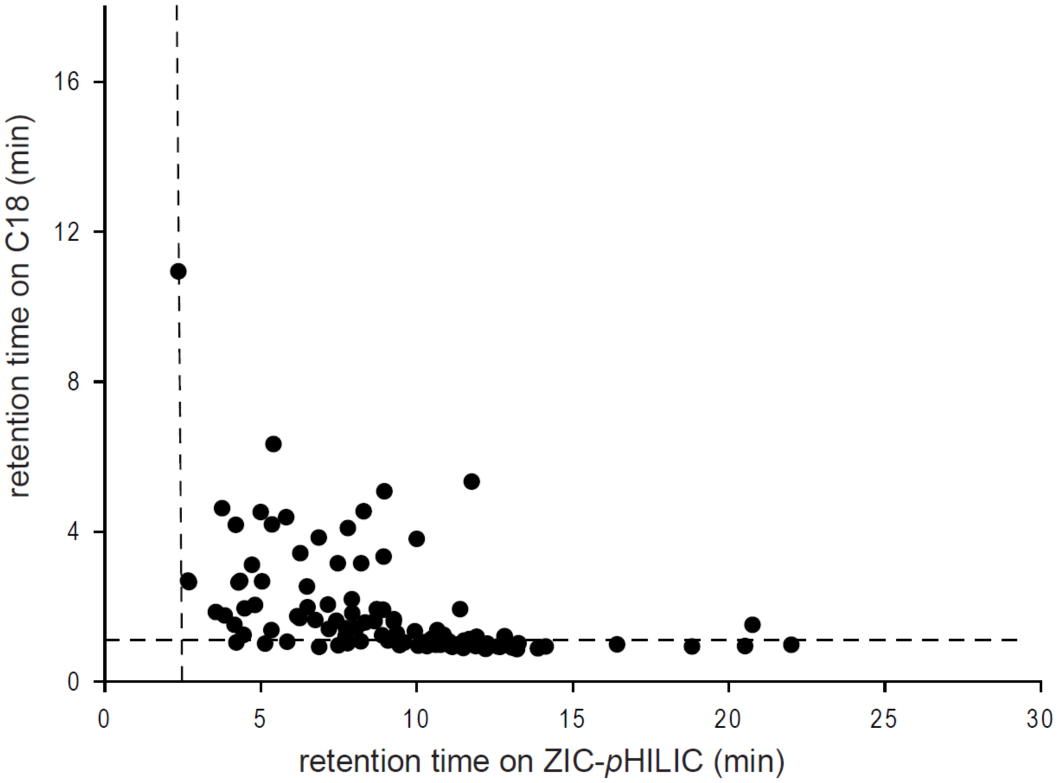

Figure S-3 Retention times for 114 detected commercial compounds representative of the bacterial metabolism on the C18 and ZIC-pHILIC column (see also Supplemental Table 5). The dotted lines represent the dead volume of each chromatographic column.

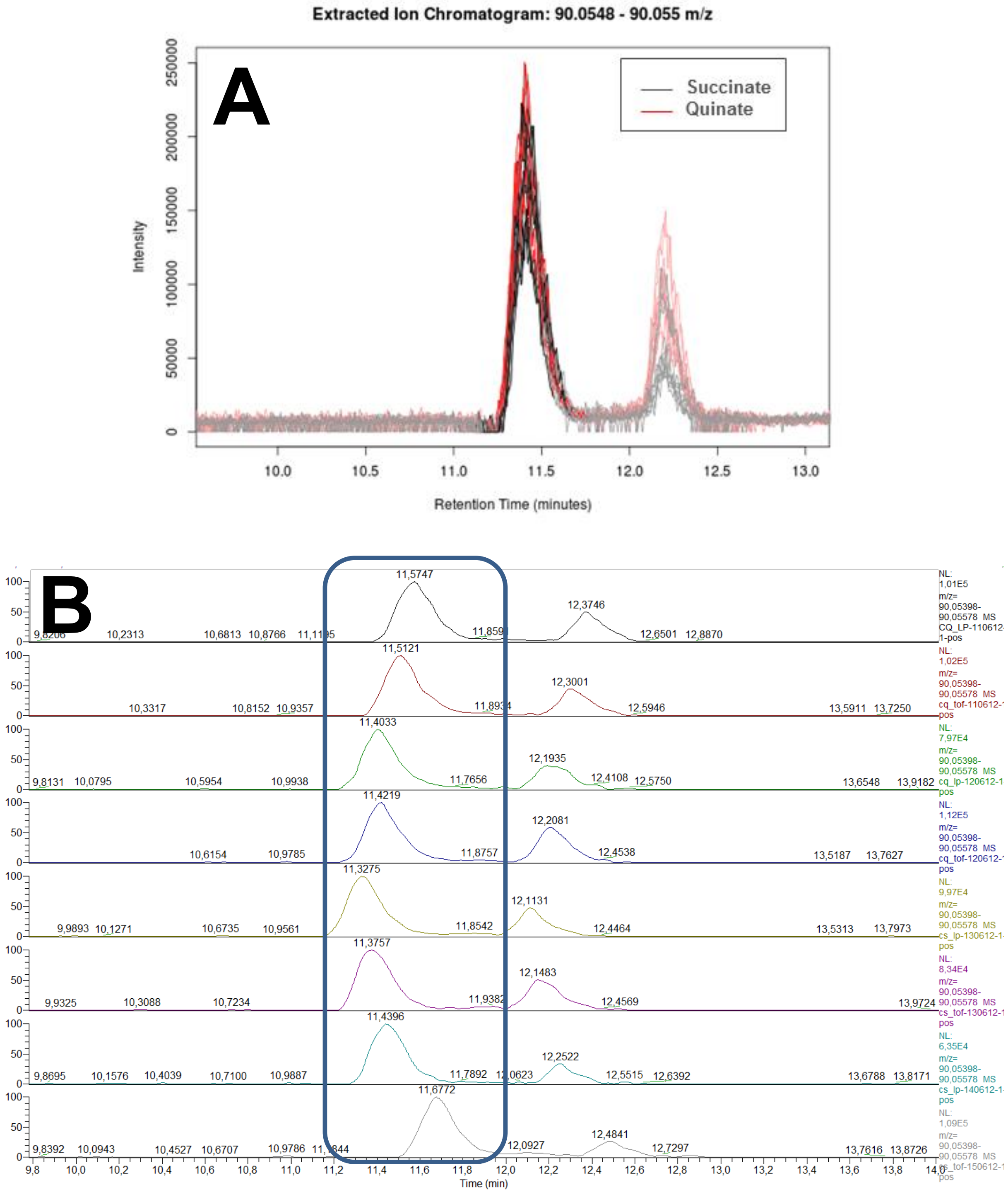

Figure S-4 Quality of XCMS data processing (peak detection, retention time alignment and fold-change) for alanine (M90T682). A: Extracted ion chromatograms from XCMS. B: Extracted ion chromatograms from RAW files. The four upper chromatograms correspond to files from the quinate group and the four bottom chromatograms correspond to files from the succinate group. Fold-change calculated by XCMS: 1.18. Fold-change calculated from peak integration in all the RAW files: 1.14.

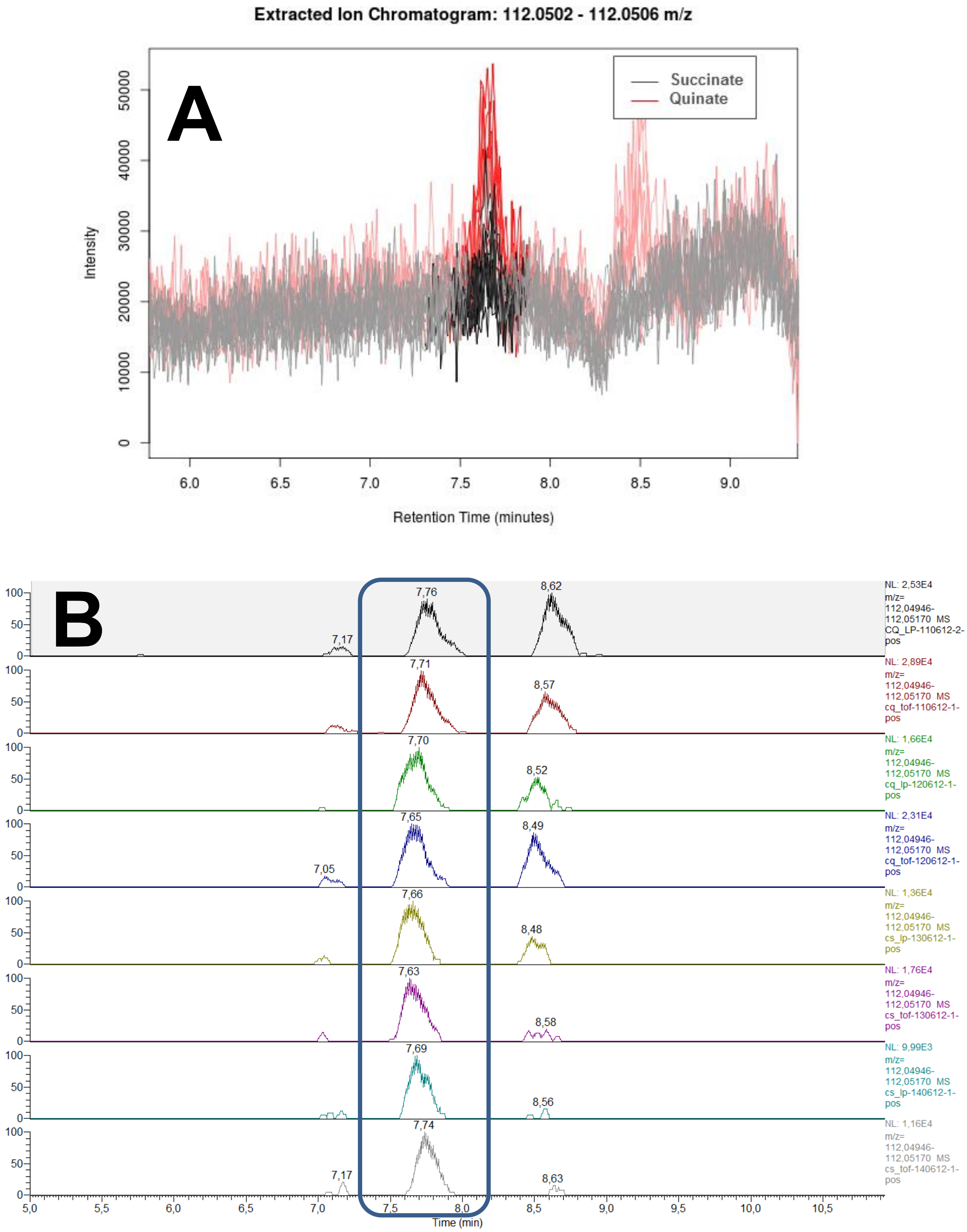

Figure S-5 Quality of XCMS data processing (peak detection, retention time alignment and fold-change) for cytosine (M112T459). A: Extracted ion chromatograms from XCMS. B: Extracted ion chromatograms from RAW files. The four upper chromatograms correspond to files from the quinate group and the four bottom chromatograms correspond to files from the succinate group. Fold-change calculated by XCMS: 1.71. Fold-change calculated from peak integration in all the RAW files: 1.62.

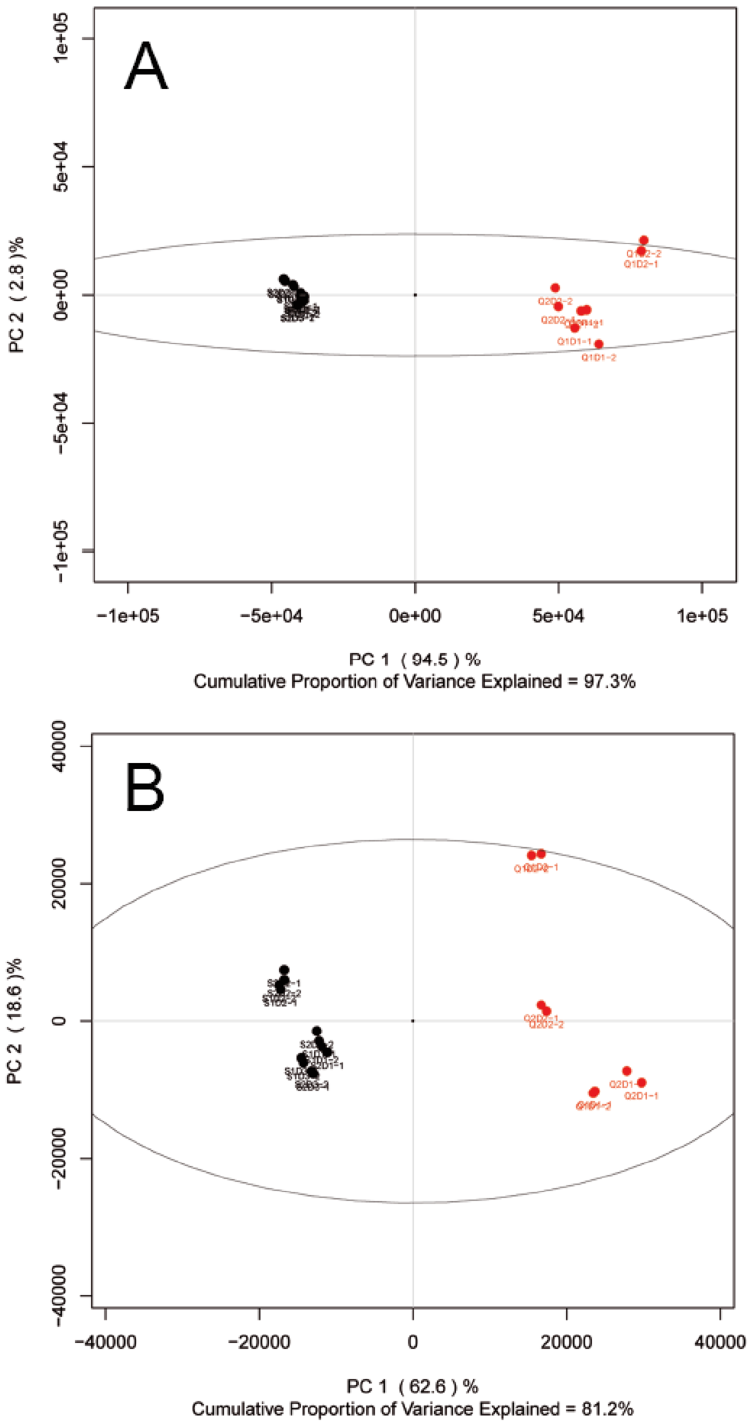

Figure S-6 PCA scores plot of the effect of the carbon source change on the metabolome of Acinetobacter baylyi ADP1. Metabolic fingerprints of ADP1 grown on succinate or quinate were recorded in negative (A) and positive (B) ionization modes. Data were mean centered and scaled to Pareto variance before multivariate statistical analysis. Quinate and succinate samples were represented by red and black circles, respectively.

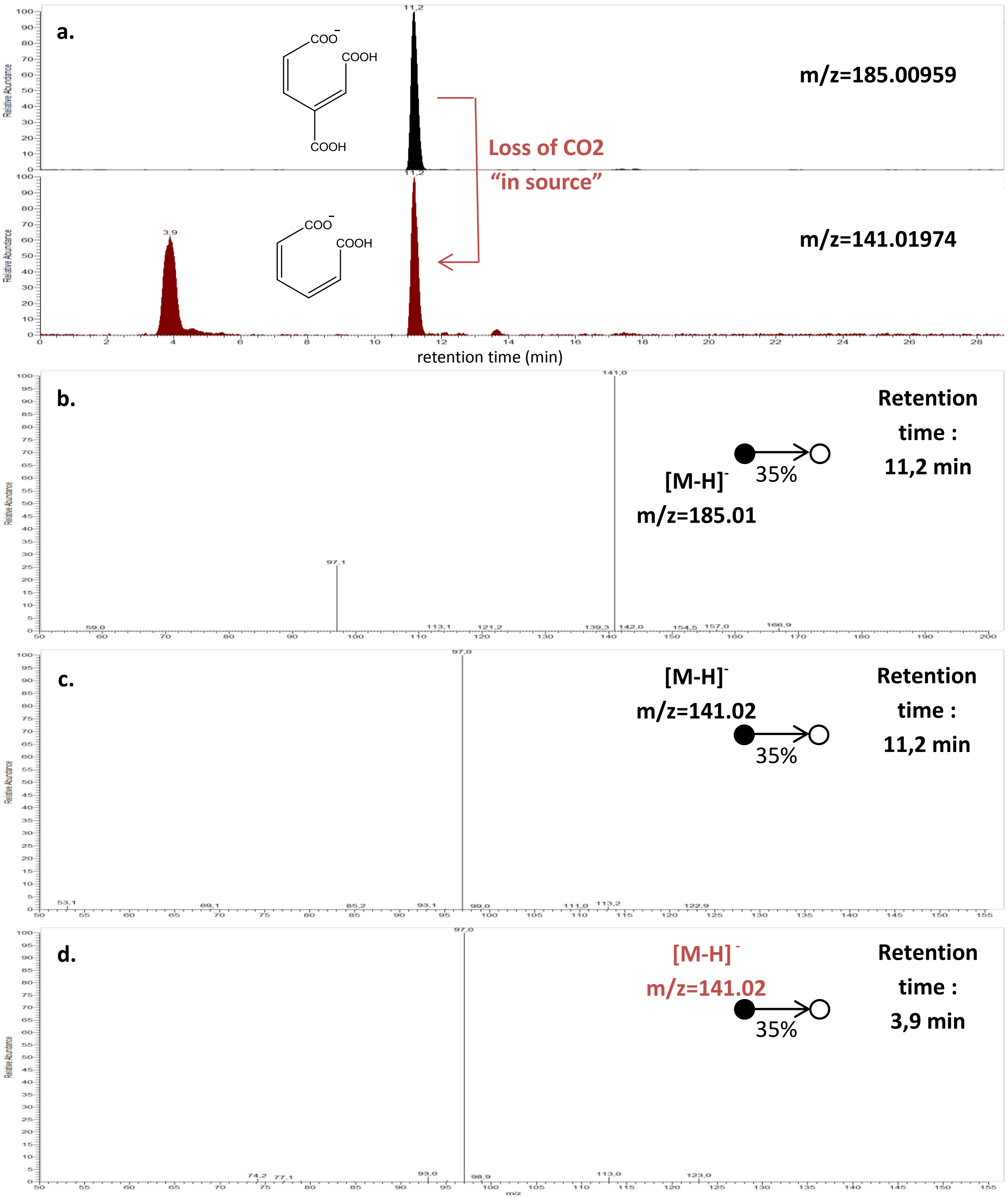

Figure S-7 Extracted ion chromatograms (XIC) and tandem mass spectra for putative 3-carboxy-cis,cis-muconic acid (expected in ADP1) and cis,cis-muconic acid (not expected in ADP1) in negative ionization mode in biological samples. **a.** XIC at the mass of 3-carboxy-cis,cis-muconic acid (185.00959) and at the mass of cis,cis-muconic acid (141.01974). **b.** MS^2 spectra from m/z 185,01 at 11,2 min. **c.** MS^2 spectra from m/z 141.02 at 11,2 min. **d.** MS^2 spectra from m/z 141.02 at 3,9 min.

At 11,2 min we observed (a.) the in-source fragmentation of the 3-carboxy-cis,cis-muconic acid in cis,cis-muconic acid (same retention time and chromatographic shape). We noticed that the MS^2 spectrum from the in-source cis,cis-muconic acid fragment (c.) was identical to the one from the peak with the same accurate mass at 3,9 min (d.). This suggested that the peak at 3,9 min was cis,cis-muconic acid.

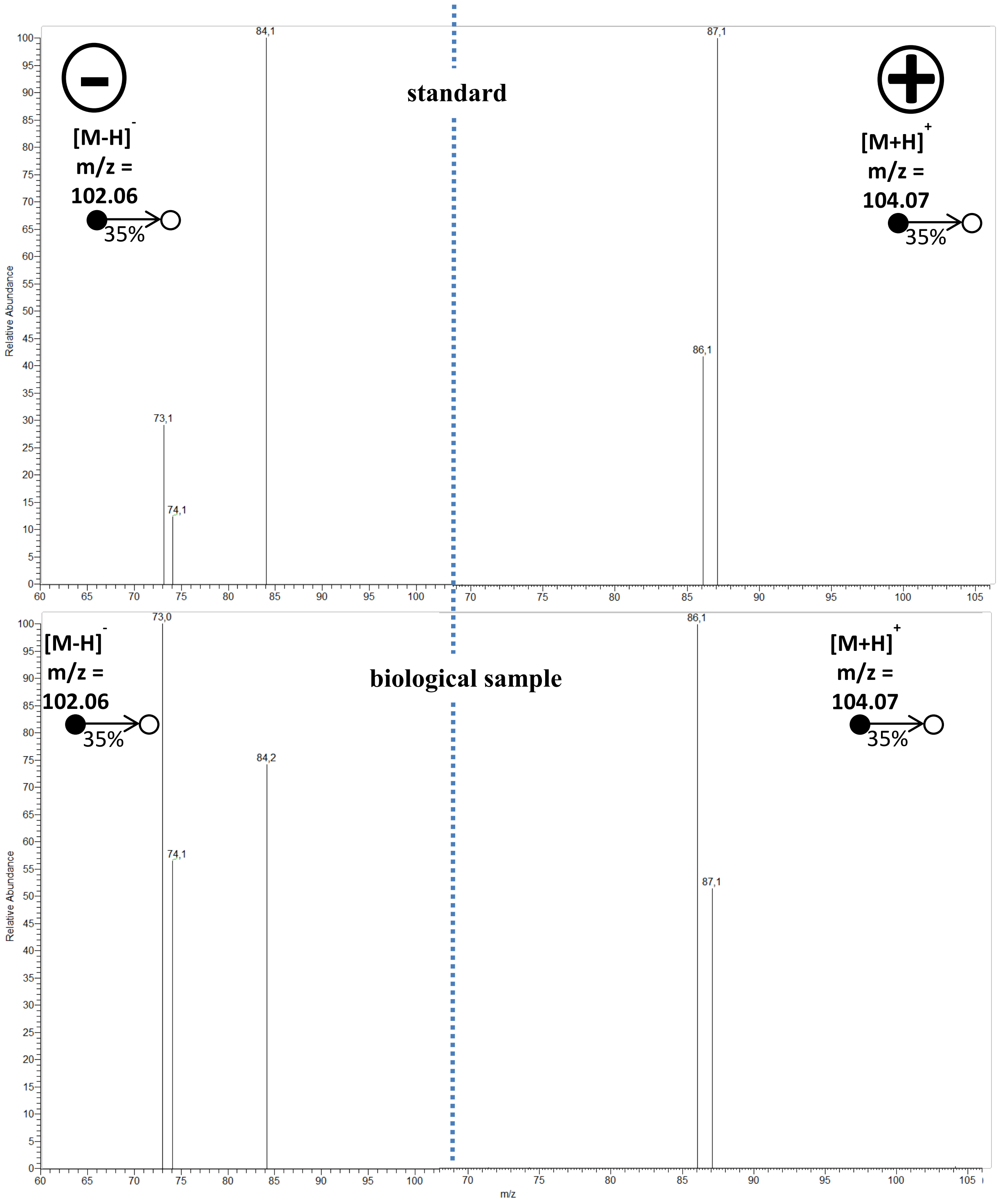

Figure S-8 Tandem mass spectrum for GABA in negative ionization mode (-) and positive ionization mode (+). The mass fragments of the reference compound were identical to those found in the biological metabolite, with slightly different relative intensities. These data were thus compatible with the presence of GABA in ADP1 metabolome.

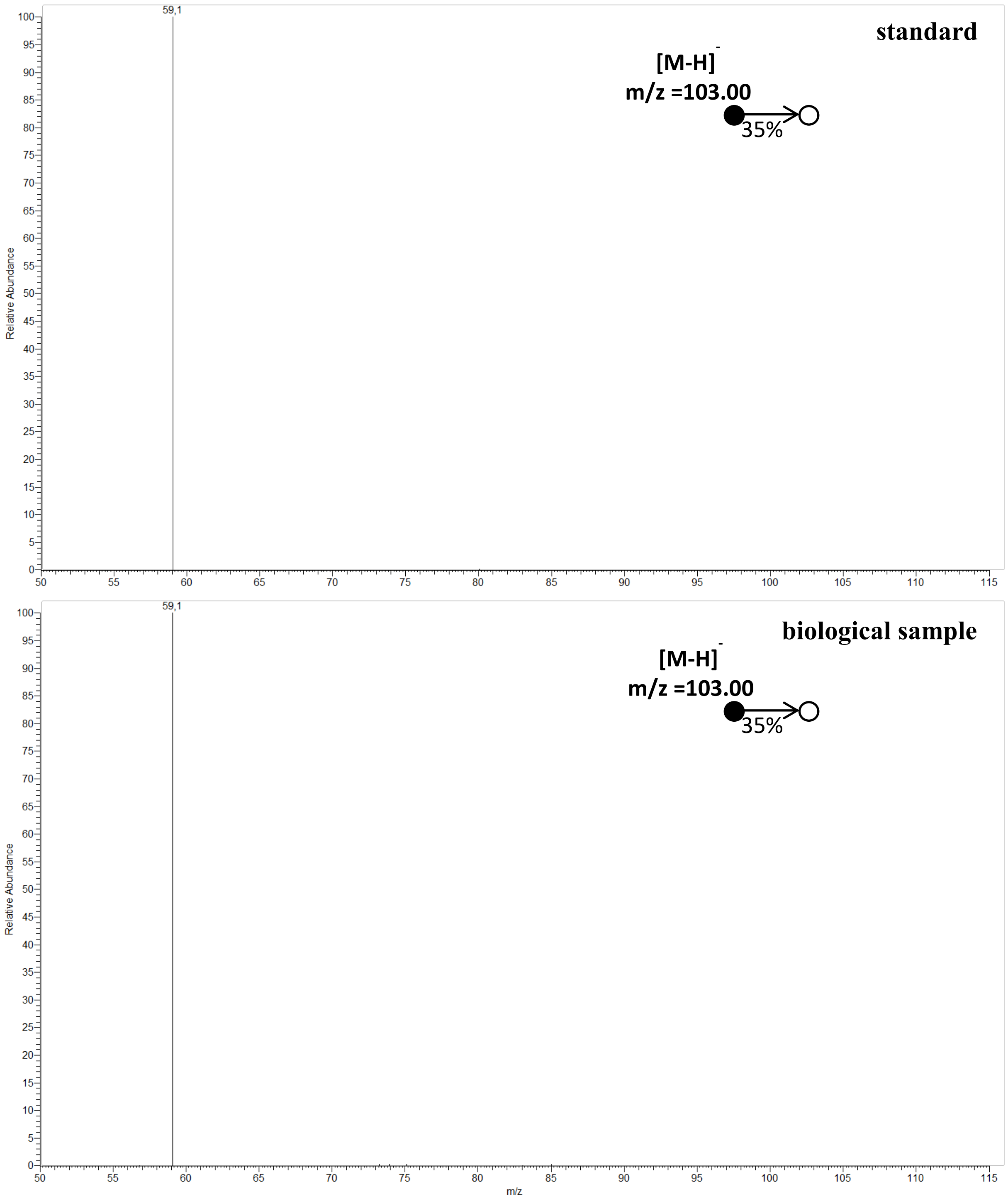

Figure S-9 Tandem mass spectrum for malonic acid in negative ionization mode. The mass of the fragment and its relative intensity were identical for the reference compound and the biological sample. These data were thus consistent with the presence of malonic acid in ADP1 metabolome.

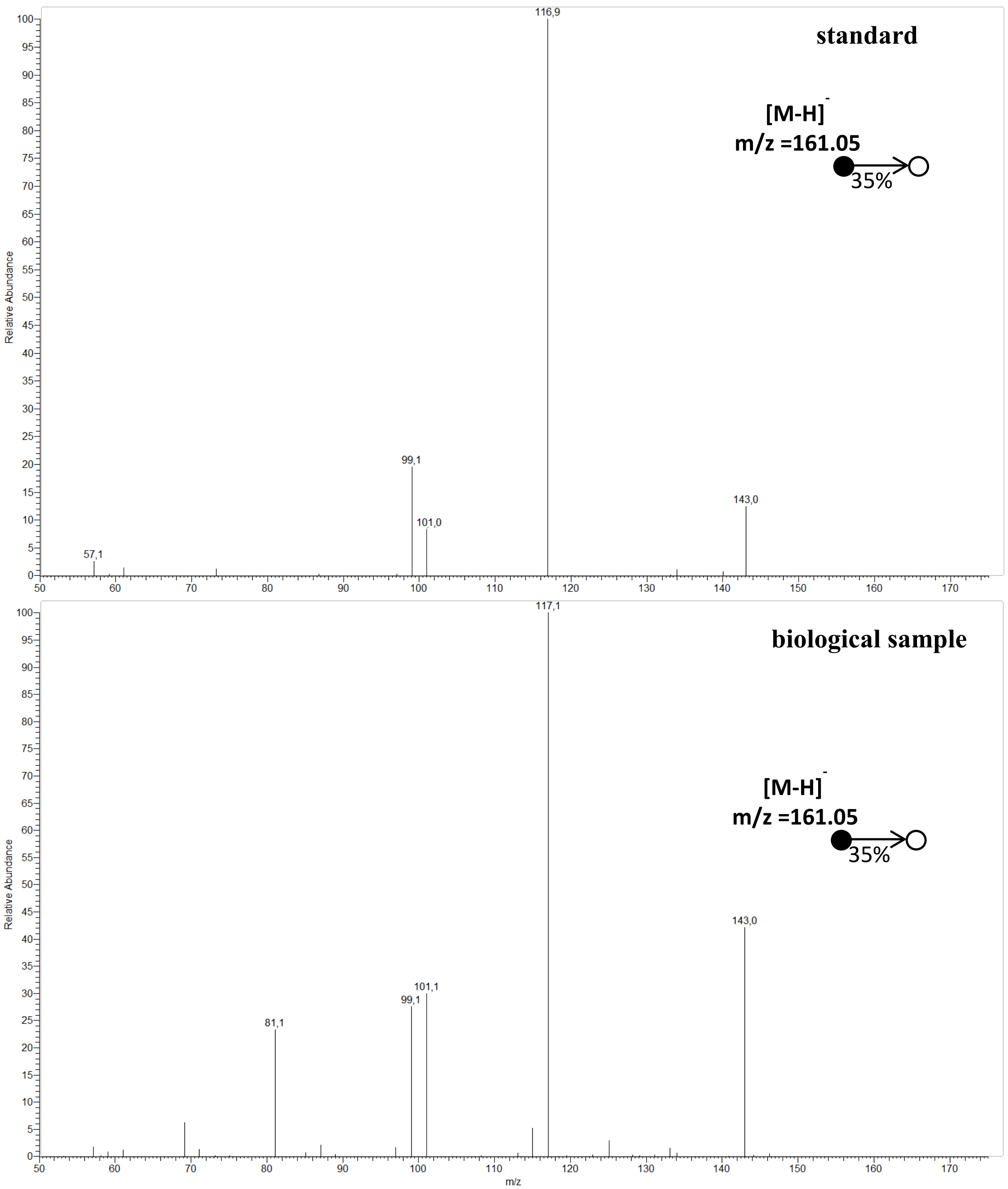

Figure S-10 Tandem mass spectrum for 3-hydroxy-3-methylglutaric acid in negative ionization mode. The fragments of the reference compounds were identified in the mass spectrum, with similar relative intensities. These data were thus consistent with the presence of 3-hydroxy-3-methylglutaric acid in ADP1 metabolome.

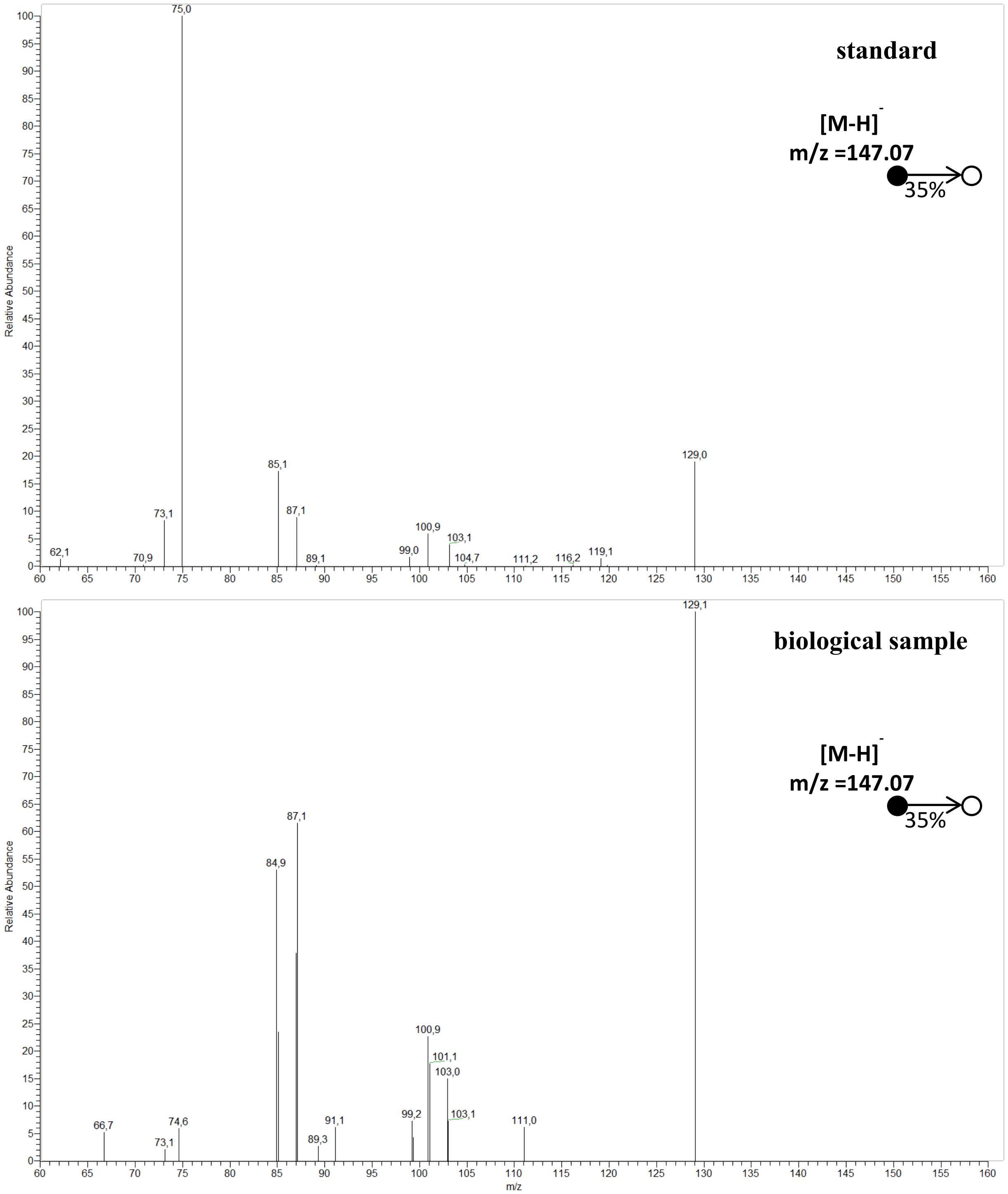

Figure S-11 Tandem mass spectrum for mevalonic acid in negative ionization mode. The main fragments of the reference compound were identified in the mass spectrum of the biological compound with slightly different relative intensities. These data were compatible with the presence of mevalonic acid in ADP1 metabolome.

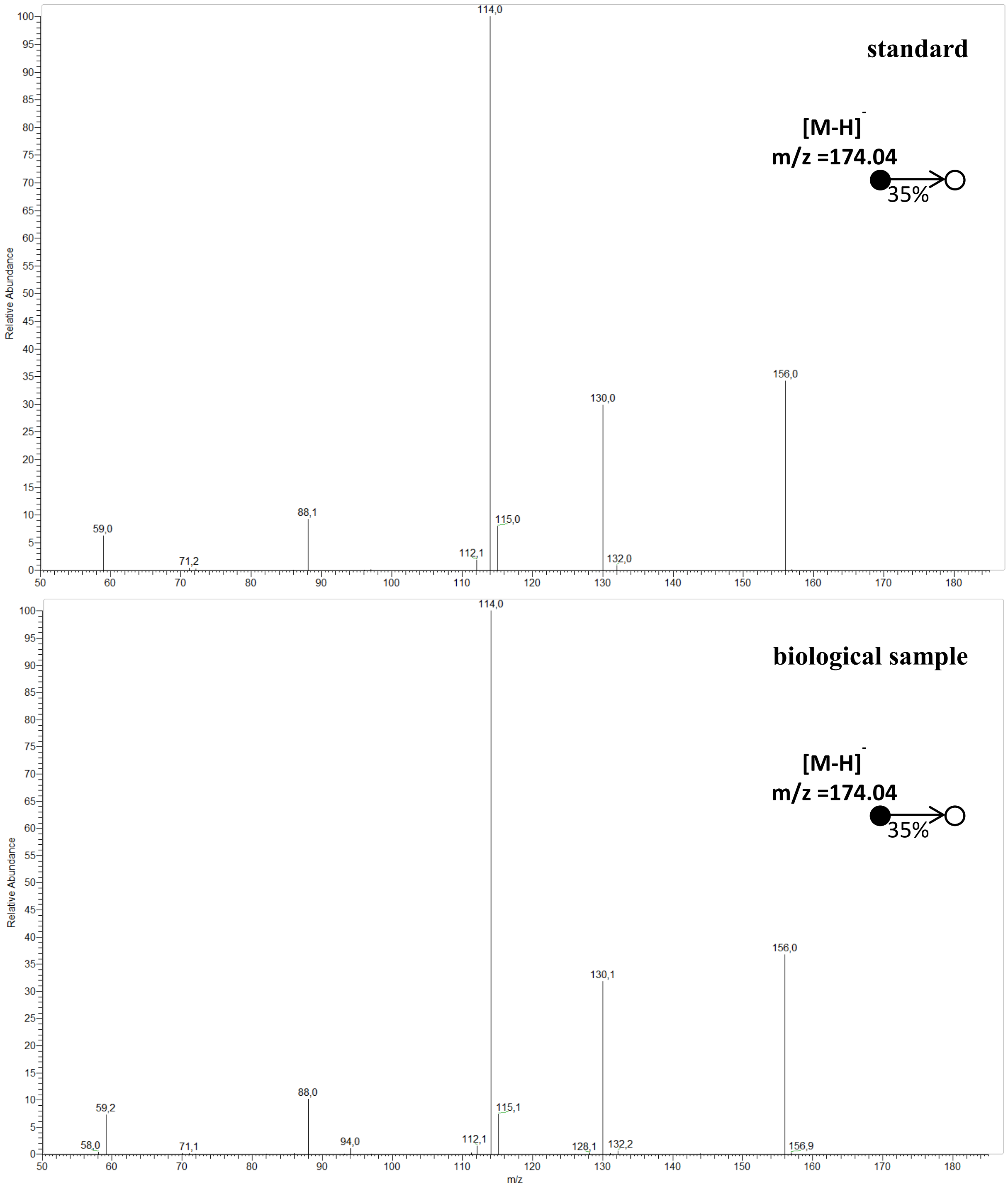

Figure S-12 Tandem mass spectrum for N-acetyl aspartic acid in negative ionization mode. The mass fragments and their relative intensities were identical in both the reference compound and the biological metabolite, suggesting the of N-acetyl aspartic acid in ADP1 metabolome.

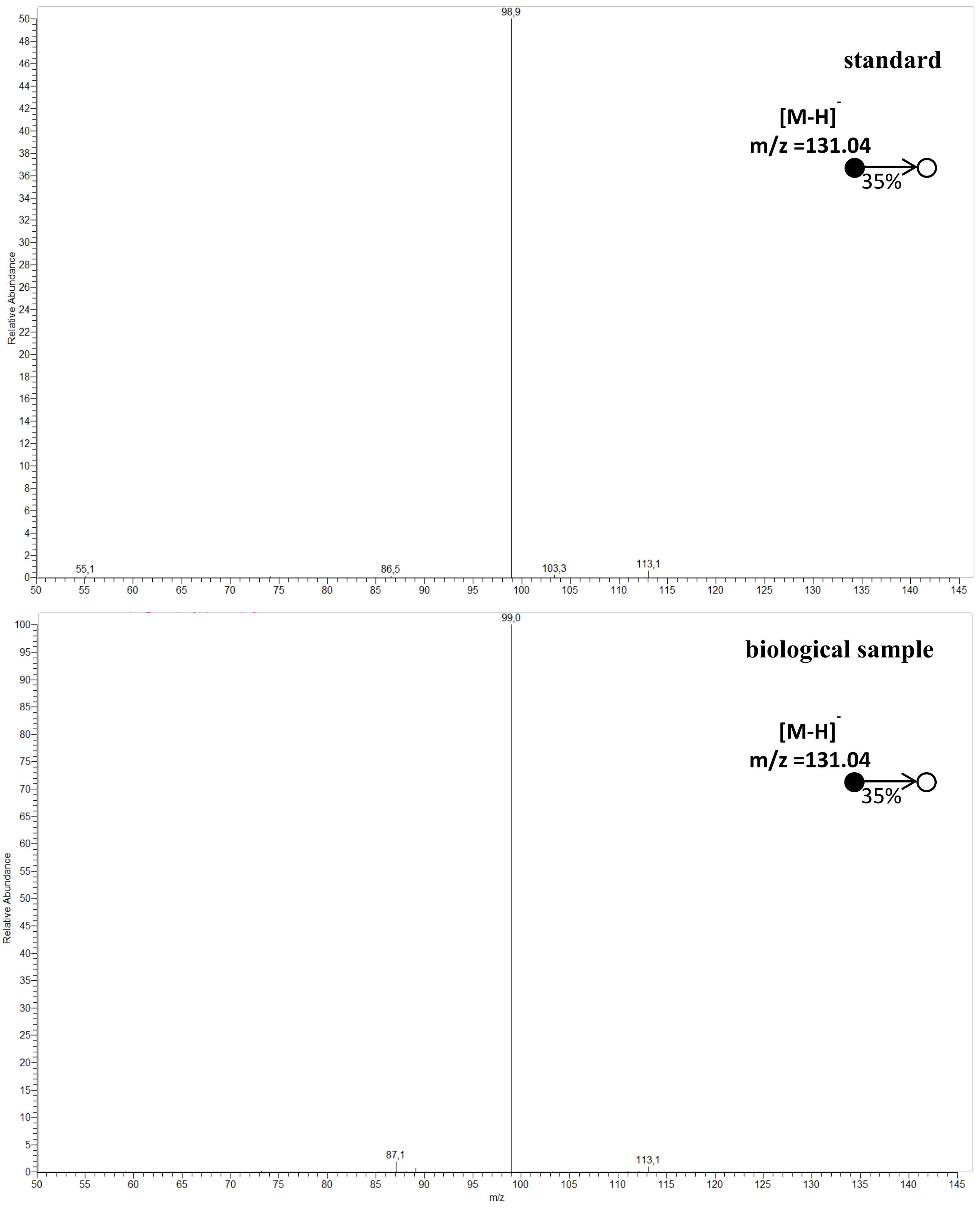

Figure S-13 Tandem mass spectrum for mono-methyl hydrogen succinic acid in negative ionization mode. The main fragments of the reference compound were identified in the mass spectrum of the biological metabolite, with identical relative intensities. These data were compatible with the presence of

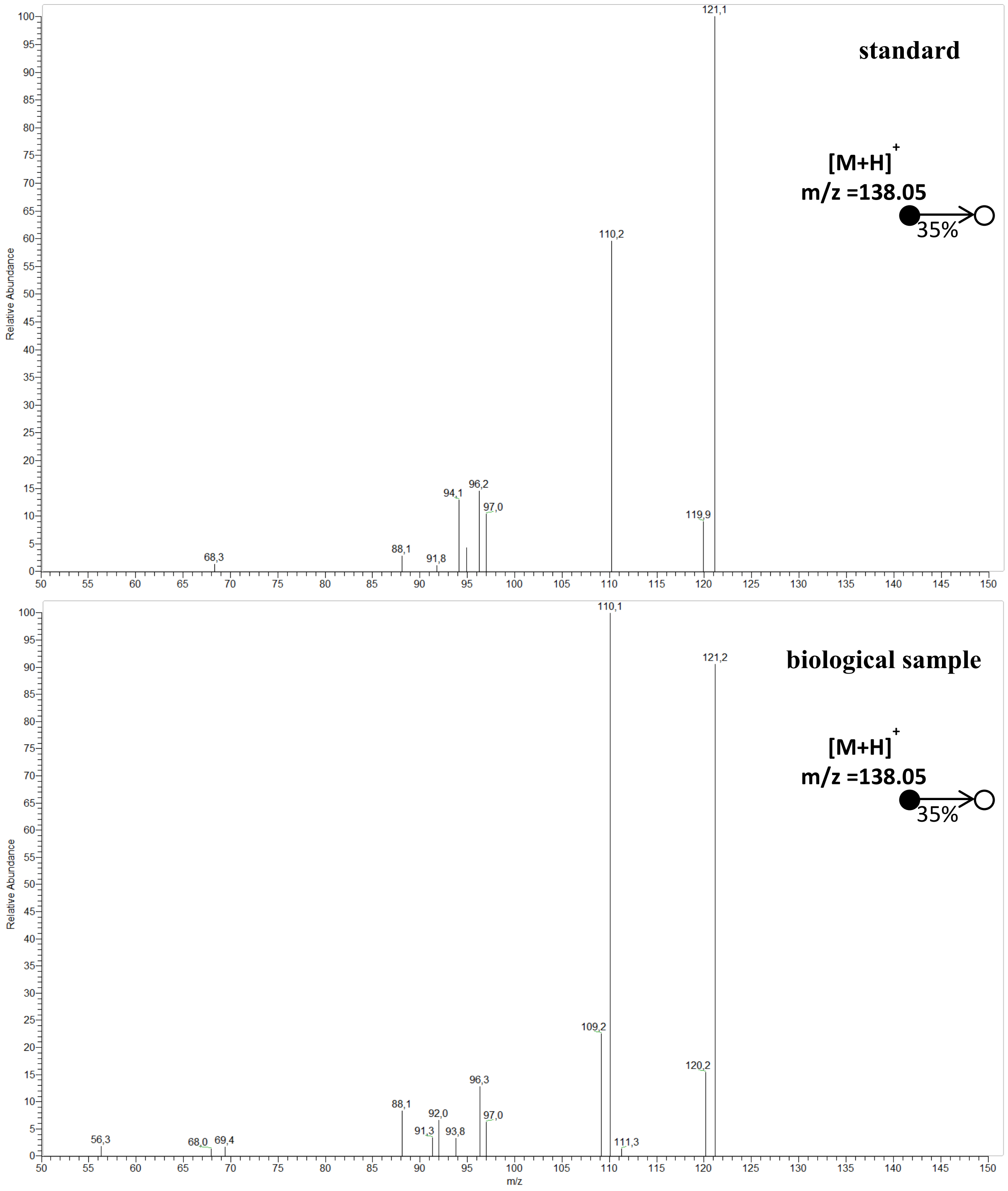

Figure S-14 Tandem mass spectrum for trigonelline in positive ionization mode. The fragments of the reference compound were identified in the mass spectrum of the biological metabolite, with identical relative intensities. These data were compatible with the presence of trigonelline in ADP1 metabolome.
